# Acknowledging uncertainty in evolutionary reconstructions of ecological niches 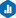


**DOI:** 10.1002/ece3.6359

**Published:** 2020-06-27

**Authors:** Hannah L. Owens, Vivian Ribeiro, Erin E. Saupe, Marlon E. Cobos, Peter A. Hosner, Jacob C. Cooper, Abdallah M. Samy, Vijay Barve, Narayani Barve, Carlos J. Muñoz‐R., A. Townsend Peterson

**Affiliations:** ^1^ Center for Macroecology, Evolution, and Climate GLOBE Institute University of Copenhagen Copenhagen Denmark; ^2^ Florida Museum of Natural History University of Florida Gainesville FL USA; ^3^ Stockholm Environment Institute Stockholm Sweden; ^4^ Department of Earth Sciences University of Oxford Oxford UK; ^5^ Biodiversity Institute University of Kansas Lawrence KS USA; ^6^ Committee on Evolutionary Biology The University of Chicago Chicago IL USA; ^7^ Entomology Department Faculty of Science Ain Shams University Cairo Egypt; ^8^ Laboratorio de Análisis Espaciales Instituto de Biología Universidad Nacional Autónoma de México Ciudad de México Mexico

**Keywords:** comparative phylogenetics, fundamental ecological niche, *Icterus*, phylogenetic niche conservatism

## Abstract

Reconstructing ecological niche evolution can provide insight into the biogeography and diversification of evolving lineages. However, comparative phylogenetic methods may infer the history of ecological niche evolution inaccurately because (a) species' niches are often poorly characterized; and (b) phylogenetic comparative methods rely on niche summary statistics rather than full estimates of species' environmental tolerances. Here, we propose a new framework for coding ecological niches and reconstructing their evolution that explicitly acknowledges and incorporates the uncertainty introduced by incomplete niche characterization. Then, we modify existing ancestral state inference methods to leverage full estimates of environmental tolerances. We provide a worked empirical example of our method, investigating ecological niche evolution in the New World orioles (Aves: Passeriformes: *Icterus* spp.). Temperature and precipitation tolerances were generally broad and conserved among orioles, with niche reduction and specialization limited to a few terminal branches. Tools for performing these reconstructions are available in a new R package called *nichevol*.

## Graphical abstract

In this manuscript, we demonstrate how the incomplete characterization of species' ecological niches affects ancestral niche reconstruction and evolutionary rate estimation, and offer a solution that incorporates uncertainty into such analyses. We first use a simple simulation to illustrate this problem and its effect on the estimation of ancestral ecological niche and rates of ecological niche evolution. We then suggest a simple, broadly useful innovation for incorporating uncertainty into fundamental niche characterization and subsequent comparative phylogenetic analyses, using an analysis of the New World orioles as a worked example.

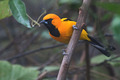

## 1 INTRODUCTION

The environmental conditions under which species thrive (i.e., their fundamental ecological niches defined in abiotic environmental dimensions) evolve over time. The frequency and speed at which niches evolve in speciating lineages remains a key question in evolutionary biology (e.g., Evans, Smith, Flynn, & Donoghue, 2009; García‐Navas & Rodríguez‐Rey, 2018; Graham, Ron, Santos, Schneider, & Moritz, 2004; Knouft, Losos, Glor, & Kolbe, 2006; Losos, 2008; Nyári & Reddy, 2013; Owens et al., 2017; Pearman, Guisan, Broennimann, & Randin, 2008; Peterson, Soberón, & Sánchez‐Cordero, 1999; Vieites, Nieto‐Román, & Wake, 2009). Methods for estimating fundamental ecological niches (Hijmans & Elith, 2015; Peterson et al., 2011) and inferring macroevolutionary patterns from phylogenies (Freckleton, Harvey, & Pagel, 2002; Lanyon, 1993; O'Meara, 2012; Pagel, Meade, & Barker, 2004; Revell, 2012; Swofford & Maddison, 1987) have both advanced greatly in recent decades. These developments have facilitated a paradigm shift toward investigating biogeographic history in the context of reconstructed ancestral ecological niche characteristics (e.g., Anciães & Peterson, 2009; Evans et al., 2009; Graham et al., 2004; Knouft et al., 2006; Nyári & Reddy, 2013; Owens et al., 2017; Pearman et al., 2008; Ribeiro, Peterson, Werneck, & Machado, 2016; Rice, Martínez‐Meyer, & Peterson, 2003; Smith & Donoghue, 2010; Vieites et al., 2009). Still, modeling complex traits and their evolution remain a major challenge, and indeed reconstructing the evolution of abiotic ecological niches is particularly difficult.

Researchers have used several approaches to characterize ecological niches when attempting to reconstruct their evolutionary history. Studies have used means and standard errors of suitable abiotic niche characteristics (Anciães & Peterson, 2009; Rice et al., 2003), minimum and maximum suitable abiotic niche values (Graham et al., 2004; Yesson & Culham, 2006), central tendencies of suitable niche values (i.e., mean or median; Ackerly, Schwilk, & Webb, 2006; Cooper, Freckleton, & Jetz, 2011; Kozak & Wiens, 2010), and distributions of suitable niche values (Evans et al., 2009; Smith & Donoghue, 2010). These data were derived either directly from the occurrence data (e.g., Ackerly et al., 2006; Cooper et al., 2011; Kozak & Wiens, 2010) or from ecological niche model outputs (e.g., Nyári & Reddy, 2013; Rice et al., 2003; Smith & Donoghue, 2010). These approaches fit existing ancestral state reconstruction methodology relatively well, but at the cost of simplifying complex niches to summary statistics for each species.

**FIGURE 1:**
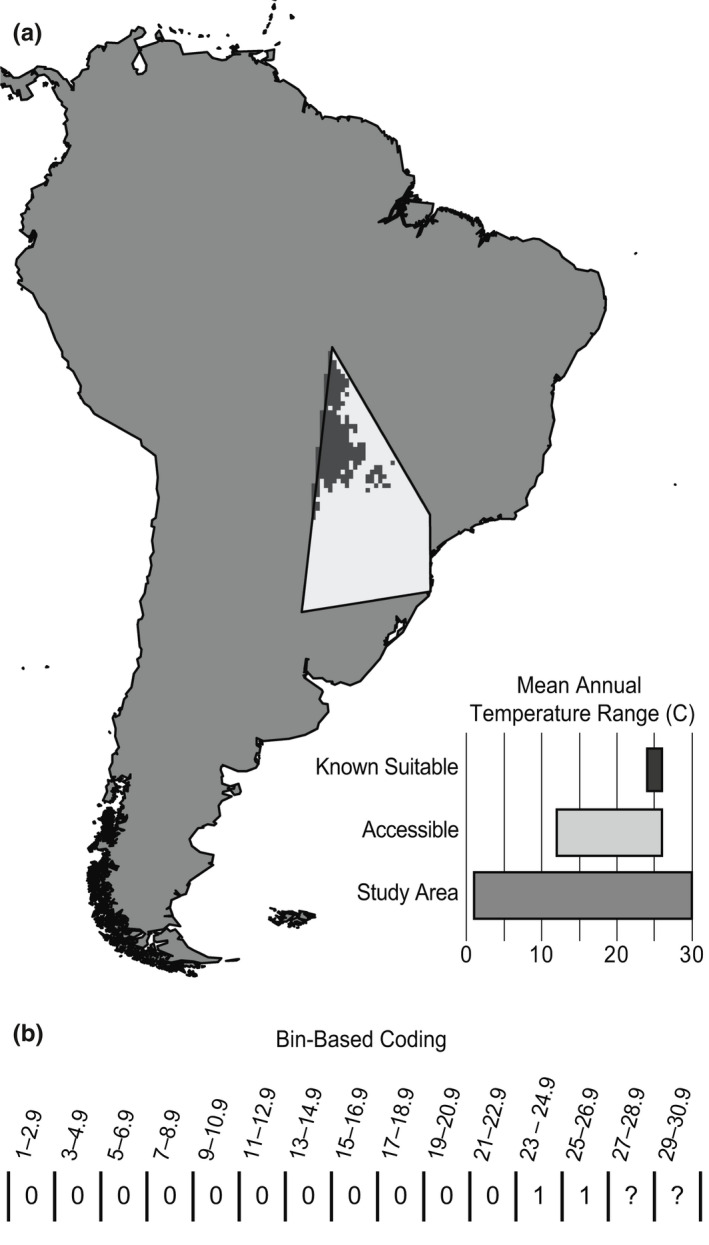
Uncertainty when characterizing fundamental niche based on realized niche. A. Map of simulation study area, distribution of suitable habitat for a simulated species (black), its accessible area (**M**; light gray), and a plot showing ranges of mean annual temperature present in each of these areas. Note that the species' lower temperature limit is found within **M**, whereas its upper limit is not. B. Example of coding abiotic niche characteristics of species mapped in Figure 1a, accounting for uncertainty. Bins with values suitable to the species are marked “1”. The lower limit is found within the species' accessible area (**M**), so coding of bins below suitable range are coded without uncertainty (“0”). The upper limit is not found within the species' accessible area (**M**), so coding of bins above known suitable range are coded as uncertain (“?”)

Fundamental ecological niches, furthermore, are rarely characterized completely and unambiguously when they are estimated for real‐world species on real landscapes, owing to biases and limitations in environmental conditions available across accessible areas of geographic space (Figure 1; Guisan, Petitpierre, Broennimann, Daehler, & Kueffer, 2014; Owens et al., 2013; Saupe et al., 2012, 2017; Veloz et al., 2012; Warren, Cardillo, Rosauer, & Bolnick, 2014). The fundamental ecological niche of a species is defined as the set of conditions under which it is able to maintain populations without immigrational input (Soberón, 2007), and is the result of phenotypic traits subject to natural selection (Peterson, 2011). However, the full suite of environmental conditions within a species' fundamental niche is not necessarily represented on Earth, or across areas that are accessible to a species. This subset of the fundamental ecological niche that is present in geographic space at the time period of interest and is accessible to the species is referred to as the existing niche (Barve et al., 2011). A species' realized ecological niche (i.e., environments where the species is observed) is determined by the further reduction of the existing niche by biotic factors such as competition and parasitism (Soberón, 2007).

As such, any characterization of fundamental ecological niches that relies on inference from species' geographic distributions (i.e., realized niche) will be incomplete (Owens et al., 2013; Saupe et al., 2012). A species' fundamental niche becomes particularly difficult to approximate from its realized niche when its geographic range approaches the limits of the area to which it can disperse (as may be the case for many island endemic species; Saupe et al., 2012). Hence, although the estimated niche of a lineage through time may show variation in response to inherited adaptations that alters the lineage's fundamental niche, that variation may also derive from changes in the set of environments accessible to that lineage, which do *not* represent a genetically inherited set of adaptations or changes in the fundamental ecological niche (Araújo et al., 2013).

Methodologies that use estimates based on species' realized niches to characterize ecological niches in phylogenetic analyses are known to overestimate true amounts of niche change (Saupe et al., 2017). Here, we present a new framework to characterize species' niches, which incorporates consideration of areas accessible to the species over relevant time periods (referred to as **M**; Soberón & Peterson, 2011; Phillips et al., 2009; VanDerWal, Shoo, Graham, & S. E. Williams SE., 2009; Barve et al., 2011;). Estimating and accounting for this accessible region has been recognized as important when generating niche or distribution models that use background or pseudo‐absence data for calibration (Barve et al., 2011; Elith, Kearney, & Phillips, 2010; Phillips et al., 2009). If regions accessible to a species are ignored when selecting the geographic extent for model calibration, fitted models may erroneously estimate suitable niche conditions. However, even niche estimates derived from presence data (i.e., without a modeling component) should consider **M**, as doing so provides one of the only ways to assess in which cases niche estimates are likely to be truncated. Specifically, when environments across **M** do not encompass conditions beyond those under which the species in question is observed, no evidence is available regarding the environmental limits of the species (Figure 1b).

**FIGURE 2:**
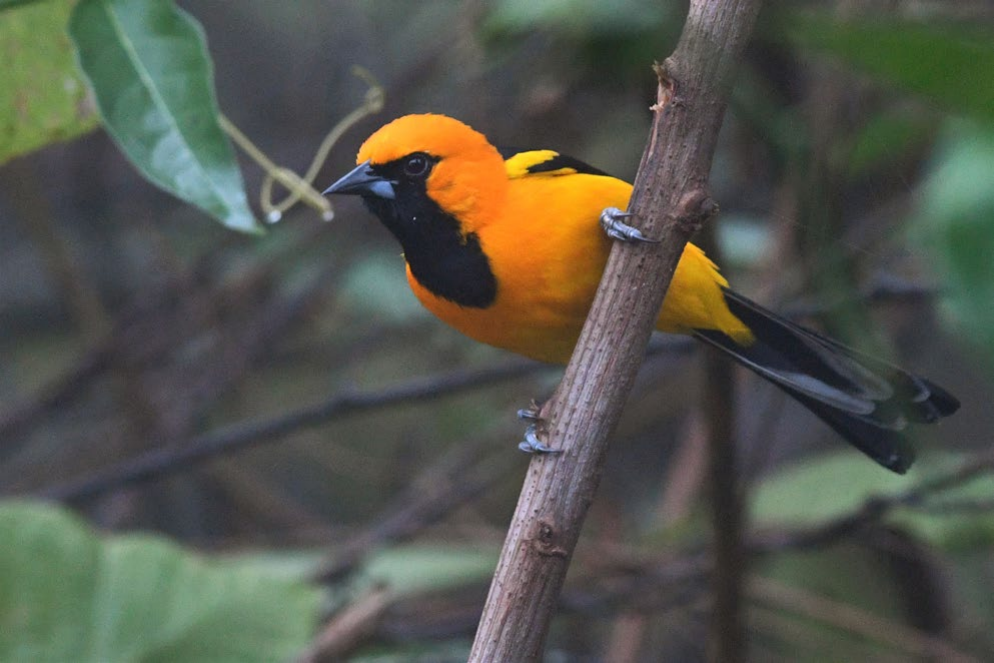
White‐edged Oriole (*Icterus graceannae*) in Macara, Ecuador (ML202492981). This striking species is endemic to the Tumbesian dry forests of northwestern Peru and southwestern Ecuador, where its specialization on drier environments is example of niche reduction identified by our new methods. Photography credit: David M. Bell

Our new binned‐range (BR) character‐coding method decomposes the broader environment occupied by and accessible to a clade into discrete bins, and scores each bin as suitable, unsuitable, or uncertain for a given species (Figure 1b), thereby accounting for potential cases of incomplete niche characterization. We illustrate the utility of summarizing species' niches in this way via simulation that compares ancestral niche reconstructions based on BR coding (Binned Ancestral Range; BAR) to those estimated using a more traditional analysis (generalized least‐squares reconstructions based on the median suitable value of a variable for each species based on its realized niche). We demonstrate the utility of our approach with an empirical example, inferring patterns of ecological niche evolution in New World orioles (*Icterus* spp.; see, e.g.*,* Figure 2). This empirical example highlights the utility of BAR reconstructions in terms of incorporating uncertainty explicitly and considering species' ecological niches as a ranged response instead of as a single value.

## 2 METHODS

### 2.1 Coding ecological niches for analysis

Coding full niche ranges represent an initial challenge for ancestral niche reconstructions. We first determine relevant analytical limits with respect to single environmental dimensions as the minimum and maximum values within the union of all accessible areas polygons for the species comprising the clade of interest. Next, we parse this range of values into equal‐width bins; for each species, bins with values falling within the range of environmental conditions occupied by the species are coded as “suitable” (1). Bins with values represented within the species' **M** but falling outside the range of environmental conditions occupied by the species are scored as “unsuitable” (0). In cases in which suitable niche conditions coincide with the limits of environmental conditions present in a species' **M**, all more extreme values—that is, values more extreme than those manifested within **M** (e.g., Figure 1)—are coded as unknown (?). This procedure allows explicit incorporation of uncertainty in our analyses. When suitability is unknown because the climatic values for the bin in question were not represented within the **M** of the species, but the bin in question was flanked by two suitable bins, it is also scored as “suitable,” under an assumption of a unimodal response to environmental conditions (Maguire, 1973). These steps can be achieved using *nichevol* v.0.1.17 (Cobos, Owens, & Peterson, 2020), an R (v.3.6.1; R Core Team, 2019) package we created to facilitate studies of niche evolution, including many of the analyses presented in this paper. Package documentation includes a tutorial demonstrating an analytical workflow implementing *nichevol*.

### 2.2 Binned ancestral range reconstruction demonstration

As a simple illustration of how this analysis works, we simulated data for a scenario of a shift from an ancestral warm niche to a cool niche, to demonstrate the ability of our new method to identify instances of niche evolution. First, we simulated distributions and accessible areas for 1,000 species across South America—500 with a fundamental mean annual temperature niche of 24–28°C (hereafter referred to as “cool‐niche species”) and 500 with a fundamental mean annual temperature niche of 25–29°C (hereafter referred to as “warm niche species”). For each set of species, accessible areas were generated using an initial population of 10,000 random polygons using *nichevol*. We assumed that each species could occupy all suitable cells within its corresponding **M** (i.e., we ignored biotic factors). Suitable cells were identified based on a 2.5′‐resolution raster of annual mean temperature data (Bio 1) from WorldClim v.1.4 (Hijmans, Cameron, Parra, Jones, & Jarvis, 2005) and the *raster* package v.3.0.0 (Hijmans, 2019) in R; simulated **M**s with no suitable conditions were removed (*n* = 1). The median suitable mean annual temperature value for each simulated species was calculated from suitable cell values within its **M**. We then determined the minimum and maximum mean annual temperature within the union of all simulated **M** polygons and parsed this range of values into equal‐width, 1°C bins using *nichevol* in R. Raw and annotated R code the niche simulation and coding steps, as well as input and output data, can be found in supplementary materials provided via Dryad (Code Supplement 1, Annotated Code Supplement 1, Data Package 1; https://doi.org/10.5061/dryad.c866t1g3j).

We generated a single, 15‐taxon stochastic birth‐death tree (birth rate = 1, death rate = 0) using the R package *phytools* v.0.6‐99 (Revell, 2012) and assigned simulated species from the cool‐niche group to a monophyletic clade of 7 taxa. The remaining tips in the tree were assigned simulated species from the warm niche simulation group. We then used *nichevol* tools to perform BAR reconstructions using maximum parsimony (as implemented in *castor* v.1.4.3; Louca & Doebeli, 2018) and maximum likelihood (as implemented in *ape* v.5.3; Paradis & Schliep, 2018). For both algorithms, ancestral state reconstructions were performed for each bin separately, treating bin scores (including “uncertain”) as discrete characters under an equal transition rate model of evolution. Results were smoothed such that reconstructed suitable ancestral niche bins at each node were not interrupted by unsuitable bins, following the assumption of a unimodal response to environmental conditions (Maguire, 1973), and accounting for evolutionary nonindependence of bins. Raw and annotated R code for these steps, as well as input and output, can be found in supplementary materials provided via Dryad (Code Supplement 2, Annotated Code Supplement 2, Data Package 1; https://doi.org/10.5061/dryad.c866t1g3j).

We note that we have kept this initial example simple for the purpose of illustration—many improvements could be made to this methodology, such as implementation of different character evolution models, Bayesian approaches in inferring ancestral character states, stochastic character mapping, and consideration of joint effects of environmental dimensions (e.g., temperature, precipitation) that are here considered independently. Furthermore, phylogenetic comparative methods are notoriously “data‐hungry,” and BAR reconstructions will benefit from further detailed simulation‐based examinations in the future. Our purpose here is to illustrate the crucial importance of incorporating uncertainty explicitly in the inference of abiotic ecological niche evolution patterns.

### 2.3 Oriole analyses

We next used BAR reconstructions to infer patterns of niche evolution in 34 species of New World orioles (genus *Icterus*). We used the single best ultrametric maximum likelihood phylogeny from Powell et al. (2014; their Figure 4) inferred from mitochondrial and nuclear DNA sequences. Distributional data for each species were drawn from the Global Biodiversity Information Facility (GBIF, 2018), a large portion of which were derived from eBird (Table S1, Data Package 2; via Dryad, https://doi.org/10.5061/dryad.c866t1g3j). We removed all records lacking geographic coordinates and inspected those remaining with respect to known ranges of species based on expert assessment by four ornithologists (authors Cooper, Hosner, and Peterson), removing records that reflected errors or outdated taxonomic arrangements. Species‐specific hypotheses of areas accessible to the species (**M**) were developed based on the biotic attributes and biogeographic history of the clade (Barve et al., 2011; Elith et al., 2010). That is, the ornithologists inspected patterns of occurrences for each species and outlined accessible area hypotheses based on known barriers to dispersal (i.e., oceans, high mountain ranges, the Amazon River, deserts). While this step remains subjective, it is crucial to a realistic representation of the environments that should be considered within the species' potential distribution (Barve et al., 2011; Phillips et al., 2009).

We then used BR to score species' niches, explicitly scoring the parts of these profiles that were not observable (i.e., at the periphery of **M**) as uncertain (see above). For mean annual temperature (Bio1 in WorldClim v.1.4; Hijmans et al., 2005), we used 32 equal‐width, 1°C bins (3–4°, 4–5°, … 34–35°) across the full range of temperature values represented in the union of all species' **M** areas. For annual precipitation (Bio12 in WorldClim v1.4; Hijmans et al., 2005), we used 80, 10‐mm‐width bins to cover the range of precipitation values from 0 to 800 mm across all species' **M** areas. For comparison to more traditional methods of coding species' niches, we calculated median values for mean annual precipitation and temperature across species' known occurrences. As with our simulated species, we characterized species' niches using R; raw and annotated R code for analyses, as well as inputs and outputs, can be found in supplementary materials provided via Dryad (Code Supplement 3, Annotated Code Supplement 3, Data Package 2; https://doi.org/10.5061/dryad.c866t1g3j).

Finally, we inferred the evolutionary history of oriole temperature and precipitation niches using both BAR, as described above, and GLS reconstructions using the median temperature and precipitation values at species occurrences. For GLS reconstructions, we first examined the fits of Brownian motion, Ornstein‐Uhlenbeck, early burst, and diffusion with linear trend models of evolution. We then performed ancestral state reconstructions using a continuous‐value maximum likelihood algorithm (as implemented in *reconstruct* in *ape*) under the best‐fit evolutionary model (Ornstein‐Uhlenbeck) for both mean annual temperature and annual precipitation. Raw and annotated R code for analyses, as well as input and output, can be found in supplementary materials provided via Dryad (Code Supplement 4, Annotated Code Supplement 4, Data Package 2; https://doi.org/10.5061/dryad.c866t1g3j).

## 3 RESULTS

### 3.1 Binned ancestral range reconstruction demonstration

Our BAR reconstructions detected simulated niche shifts; maximum likelihood reconstructions performed more reliably than parsimony reconstructions. In our simulated example, using maximum likelihood, we were able to recover the expansion from a 25°C ancestral lower fundamental niche limit to a 24°C ancestral lower fundamental niche limit at the most recent common ancestor of the 7 cool‐niche simulated species (Annotated Code Supplement 1). However, the parsimony‐based reconstruction failed to recover this change fully, but did show an expansion to 24°C for simulated species “t1,” a species with a higher maximum known suitability than the other warm niche species (Annotated Code Supplement 1). By comparison, the GLS reconstruction performed qualitatively worse, reconstructing shifts to a *warmer* niche for the simulated cool‐niche species and their ancestors. This is likely due to biased estimates of species' realized niches based on their existing niches—“t3,” a cool‐niche species, had a median suitable temperature of 26.6°C, tied for the highest temperature in the clade with “t5,” a simulated warm niche species. Interestingly, BAR using parsimony reconstruction tended to infer more uncertain character states at the cooler ends of ancestral niches, whereas BAR using ML reconstruction inferred more uncertain character states at the warmer ends of ancestral niches. See Annotated Code Supplement 1 for further detail.

### 3.2 Application to oriole niche evolution

Large numbers of occurrence points were available for this clade, thanks to recent advances in biodiversity informatics and community‐science initiatives regarding bird distributions (Table S1, Data Package 2). Niche estimates for some oriole species were completely characterized with respect to **M**, including the temperature and precipitation dimensions for *Icterus fuertesi*, and the precipitation dimensions for *I. graceannae* and *I. galbula* (Figures S2 and S3). That is, estimated limits of suitable conditions were contained completely within the environments available in **M** and did not appear to be truncated. The majority of species, however, were estimated to have niche ranges flanked by unknown maxima and/or minima.

**FIGURE 3:**
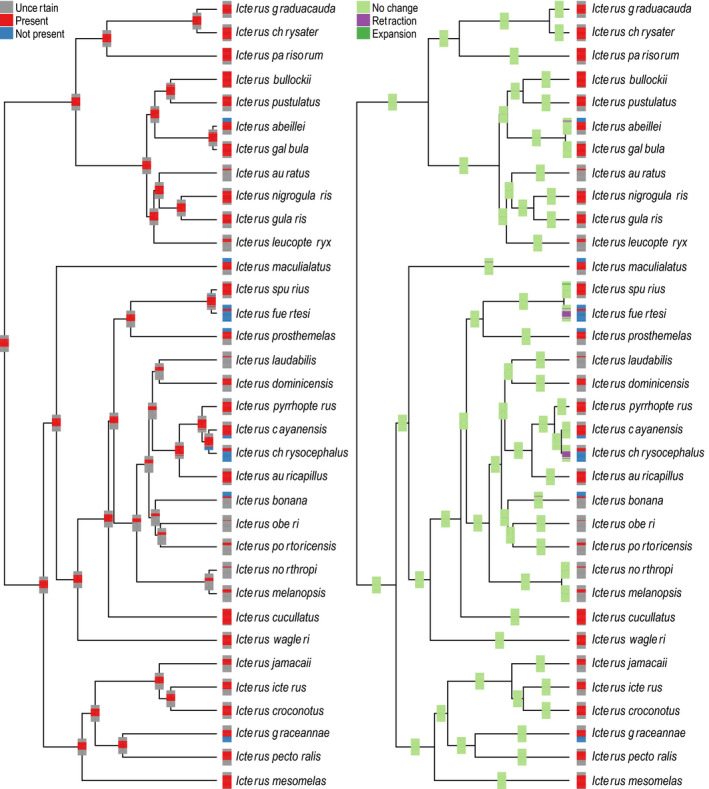
New World oriole (*Icterus* spp.) mean annual temperature niche state inference, characterized using bins and reconstructed with maximum likelihood, showing general niche conservatism. Left panel shows bin‐based characterization of niches at tips and reconstructed bin‐based values at nodes; bars show bin coding from highest bin at top to lowest bin at bottom. Right panel shows bin‐based characterization of niches at tips, comparison with ancestors at nodes. See Annotated Code Supplement 4 for maximum parsimony reconstructions (Dryad https://doi.org/10.5061/dryad.c866t1g3j)

**FIGURE 4:**
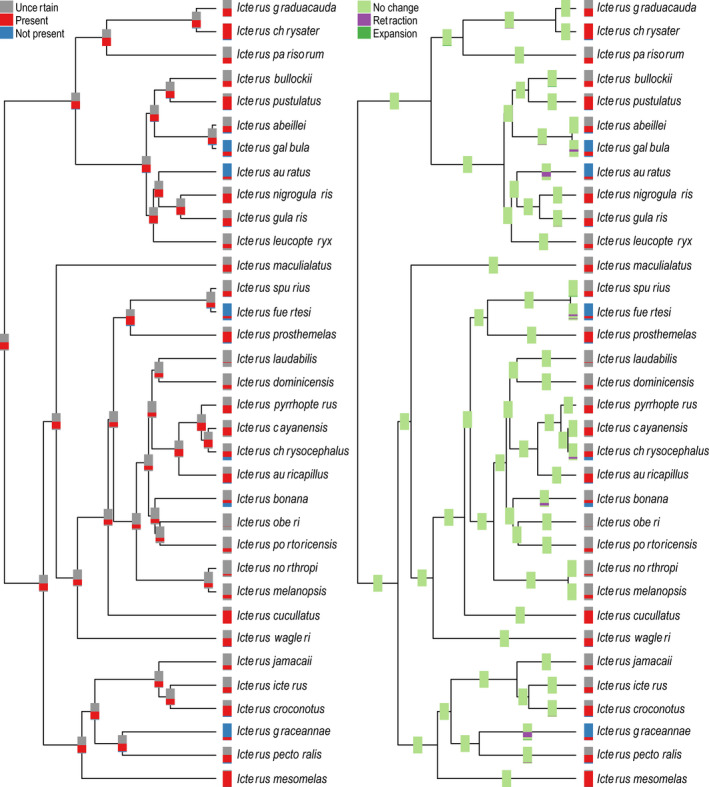
New World oriole (*Icterus* spp.) annual precipitation niche state inference, characterized using bins and reconstructed with maximum likelihood, showing general niche conservatism. Left panel shows bin‐based characterization of niches at tips and reconstructed bin‐based values at nodes; bars show bin coding from highest bin at top to lowest bin at bottom. Right panel shows bin‐based characterization of niches at tips, comparison with ancestors at nodes. See Annotated Code Supplement 4 for maximum parsimony reconstructions (Dryad https://doi.org/10.5061/dryad.c866t1g3j)

In general, BAR reconstructions of species' ecological niches in *Icterus* did not recover reduction or gain in inferred suitable niche space, suggesting broad‐scale evolutionary stability (Figures 3 and 4; Tables S2‐S5, Annotated Code Supplement 4). For temperature, both ML and parsimony BAR reconstructed a consistent range of mean annual temperature niche values across all ancestral nodes (hereafter referred to as a “core conserved niche”), although some individual nodes had lower minimum or higher maximum suitable temperatures; the estimated core conserved niche was much broader for maximum likelihood (21–26°C) than parsimony (24–25°C; Tables S2 and S3). For precipitation, maximum likelihood reconstructed a core conserved annual precipitation niche range of 71–240 mm, whereas parsimony‐based reconstructions recovered no clear core conserved niche for precipitation (Tables S4 and S5).

Because GLS and BAR reconstructions generate quite different outputs, direct comparisons are not possible; however, qualitatively, single‐value reconstructions appear to evolve much more quickly than bin‐based reconstructions, especially near the base of the tree and in groups with a preponderance of island endemics. GLS reconstructions based on median environmental values fell within ranges of values inferred using the bin‐based method at every node for both environmental variables (Tables S3 and S5). However, these reconstructions were only congruent with maximum parsimony BAR reconstructions for 24 of 33 nodes for mean annual temperature, and for none of the nodes for annual precipitation (Tables S2 and S4).

## 4 DISCUSSION

### 4.1 New methodology

This contribution derives from careful examination and analysis of the growing suite of papers analyzing niche evolution across phylogenies (e.g., Evans et al., 2009; Knouft et al., 2006; Losos, 2008; Meseguer, Lobo, Ree, Beerling, & Sanmartín, 2015; Nyári & Reddy, 2013; Peterson et al., 1999; Vieites et al., 2009; Wiens & Graham, 2005). It is likely that fundamental niches and realized niches are rarely equivalent, owing to constraints imposed by the set of environments that can be observed within areas accessible to a species (**M**) (Soberón & Peterson, 2011). The limited environments present in areas accessible to species typically will add variation to niche estimates that will bias analyses of niche evolution toward concluding increased niche lability (Ribeiro et al., 2016; Saupe et al., 2017). In addition, use of summary statistics to characterize species' niches introduces further variation related to the environmental vagaries of sampling, which has its own intrinsic biases (Kadmon, Farber, & Danin, 2004) that are—again—reflected in the environmental signature of the occurrence data that derive from the process (Saupe et al., 2017).

Analyzing ecological niche change on a phylogenetic tree without considering uncertainty produces more concise conclusions and is easier to implement (e.g., calculating the median or mean of environmental values across all occurrences for a species and performing a single reconstruction calculation). However, previous studies indicate that this approach comes with a cost: Niche change may be over‐ or under‐estimated, introducing biases in reconstructing evolutionary change in niches through time (Ribeiro et al., 2016; Saupe et al., 2017). Our empirical example using orioles shows patterns that are qualitatively consistent with these findings: GLS reconstructions of ancestral node characteristics varied more near the base of the tree and in clades dominated by narrow‐range endemic species with incompletely characterized abiotic ecological niches than in clades with fewer narrow‐range endemics.

Admittedly, we currently lack a method for quantitative assessment of niche evolution rates estimated from BAR reconstructions that is comparable to rates calculated for single continuous value reconstructions (especially in light of differences in how evolutionary models are applied and how rates are estimated for continuous and discrete characters). However, our BAR reconstructions appear to be qualitatively more robust to noise in the data introduced by narrow‐range endemics that are incompletely characterized, as it recovered conserved ranges of suitable habitat for all basal *Icterus* lineages (except for parsimony reconstructions of precipitation). Furthermore, BR coding is less likely to be skewed by instances of biased sampling, (i.e., a greater frequency of occurrences within a particular environmental range can skew niche estimates based on summary statistics). Indeed, a greater abundance of occurrences in particular environments may not be due to those environments being more suitable to a species than another suite of environments, but merely that those environments are more common within a species' **M** or more likely to be sampled by researchers. This is illustrated by our simulation reconstruction, in which the median ancestral temperature for the cool‐niche simulated species was inferred to be *warmer* owing to biased tip state characterizations.

### 4.2 Oriole niche evolution

The genus *Icterus* exists in many different environments, which suggests that the niches of these species have diversified. Indeed, when we look at patterns of niche evolution inferred using GLS, we found frequent apparent niche shifts across the phylogeny, particularly within clades dominated by island endemics (Annotated Code Supplement 4, Tables S2‐S5). However, BAR reconstructions found little evidence of change in the inferred fundamental ecological niche across the phylogenetic history of the genus, particularly when reconstructions were done using the maximum likelihood algorithm. This pattern is consistent with the fact that species of *Icterus* that “left” the Tropics (i.e., migratory species) move into northern areas of North America in the breeding season only—a special case of niche conservatism termed “niche following” in previous work (Joseph & Stockwell, 2000; Nakazawa, Peterson, Martínez‐Meyer, & Navarro‐Sigüenza, 2004).

The overall tendency across the history of the genus *Icterus* was one of remarkable niche stability, notwithstanding the GLS results. Particularly, invariant was the upper end of the temperature tolerance spectrum (Figure 3; Figure S2, Tables S2 and S3; Annotated Code Supplement 4). This observation coincides with recent results from Araújo et al. (2013), who presented a meta‐analysis that concluded that heat tolerance was much more constrained over evolutionary history than cold tolerance. Importantly, though, our proposed framework for characterizing ecological niches and subsequent ancestral niche inference may underestimate true amounts of niche evolution because the method only concludes niche change when explicit evidence exists, which we consider to be a desirable quality. Still, further detailed simulation study is needed to examine fully the sensitivity of our proposed methods to true niche evolution in the face of various biasing or obfuscating factors.

Focusing on maximum likelihood BAR reconstructions, which showed clearer patterns with less uncertainty than parsimony BAR reconstructions, we identified niche reductions for species that are relative habitat specialists within *Icterus*. *Icterus* orioles are a predominately lowland group, although some species occur in foothills and low montane regions adjacent their core lowland ranges. We identified reductions in high temperature tolerance for two species that specialize in Mesoamerican montane habitats, *I. abeillei* and *I. maculialatus*, and reductions in low temperature tolerance for two strictly lowland tropical species, *I. fuertesi* and *I. chrysocephalus. Icterus* orioles occupy a variety of forest types across a variety of precipitation regimes. However, for two species that specialize in dry forest, *I. auratus* of the Yucatán Peninsula and *I. graceannae* of the Tubezian region, we identified suitable niches corresponding to reduced precipitation.

## 5 CONCLUSIONS

The challenge of understanding change in species' ecological niches across evolutionary history lies in characterizing the entirety of a species' niche. We present a simple methodology that directly incorporates knowledge gaps based on incomplete niche characterization. We see a number of next steps in developing this methodology further—specifically, developing *nichevol* tools to encompass Bayesian estimation approaches and considering alternative evolutionary models. We would also take into account the frequency of occurrence of environmental conditions across the accessible area of each species in making conclusions about niche limitations (e.g., Meyer & Pie, 2018)—that is, non‐occurrence in relatively rare environments should perhaps not be taken as evidence of niche limitation. Finally, we plan to develop a method for estimating the likely range of niche evolution rates encompassing uncertainty using our bin‐based method. We are exploring implementation of these next steps in coming applications of this methodology.

## CONFLICT OF INTEREST

None declared.

## AUTHOR CONTRIBUTIONS

**Hannah L. Owens:** Conceptualization (equal); data curation (equal); formal analysis (equal); investigation (equal); methodology (equal); project administration (lead); resources (equal); software (equal); supervision (equal); Validation (equal); visualization (equal); writing–original draft (equal); writing–review and editing (equal). **Vivian Ribeiro:** Conceptualization (equal); data curation (equal); formal analysis (equal); investigation (equal); methodology (equal); resources (equal); software (equal); validation (equal); visualization (equal); writing–original draft (equal); writing–review and editing (equal). **Erin E. Saupe:** Conceptualization (equal); data curation (equal); formal analysis (equal); investigation (equal); methodology (equal); resources (equal); supervision (equal); validation (equal); visualization (equal); writing–original draft (equal); writing–review and editing (equal). **Marlon E. Cobos:** Data curation (equal); formal analysis (equal); investigation (equal); methodology (equal); resources (equal); software (equal); supervision (equal); validation (equal); visualization (equal); writing–review and editing (equal). **Peter A. Hosner:** Conceptualization (equal); data curation (equal); formal analysis (equal); investigation (equal); methodology (equal); resources (equal); supervision (equal); validation (equal); visualization (equal); writing–original draft (equal); writing–review and editing (equal). **Jacob C. Cooper:** Conceptualization (equal); data curation (equal); formal analysis (equal); investigation (equal); methodology (equal); resources (equal); supervision (equal); validation (equal); visualization (equal); writing–original draft (equal); writing–review and editing (equal). **Abdallah M. Samy:** Conceptualization (equal); data curation (equal); formal analysis (equal); investigation (equal); methodology (equal); resources (equal); supervision (equal); validation (equal); visualization (equal); writing–original draft (equal); writing–review and editing (equal). **Vijay Barve:** Conceptualization (equal); data curation (equal); formal analysis (equal); investigation (equal); methodology (equal); resources (equal); software (equal); supervision (equal); validation (equal); visualization (equal); writing–original draft (equal); writing–review and editing (equal). **Narayani Barve:** Conceptualization (equal); data curation (equal); formal analysis (equal); investigation (equal); methodology (equal); resources (equal); software (equal); supervision (equal); validation (equal); visualization (equal); writing–original draft (equal); writing–review and editing (equal). **Carlos J. Muñoz‐R.:** Conceptualization (equal); data curation (equal); formal analysis (equal); investigation (equal); methodology (equal); resources (equal); supervision (equal); validation (equal); visualization (equal); writing–original draft (equal); writing–review and editing (equal). **A. Townsend Peterson:** Conceptualization (equal); data curation (equal); formal analysis (equal); investigation (equal); methodology (equal); project administration (supporting); resources (equal); software (equal); supervision (equal); validation (equal); visualization (equal); writing–original draft (equal); writing–review and editing (equal).

### Open Data Badge

This article has earned an Open Data Badge for making publicly available the digitally‐shareable data necessary to reproduce the reported results. The data is available at https://doi.org/10.5061/dryad.c866t1g3j.

## Supporting information

Appendix S1

## Data Availability

Analysis scripts, annotate HTML script reports, **M** polygons for both the virtual species and empirical case, final oriole occurrence datasets, and results of niche characterization are accessible via Dryad (https://doi.org/10.5061/dryad.c866t1g3j). Pre‐acceptance private reviewer link available via https://datadryad.org/stash/share/‐RSOGe75ToJPMS7mS2c7S4SQOGODISY_ecgq7p7ovuA.
