# Supplementary material for: Acknowledging uncertainty in evolutionary reconstructions of ecological niches [image]
Source: Ecol Evol. 2020 Jun 27;10(14):6967–77. doi: 10.1002/ece3.6359 (PMC7391559; doi:10.1002/ece3.6359)
Supplement: Supplementary file 1 — Appendix S1 [file ECE3-10-6967-s001.pdf]

Reconstructing Ecological Niche Evolution via Ancestral State Reconstruction with Uncertainty Incorporated

Supplementary Tables and Figures

Supplementary Figure 1. Phylogeny of *Icterus* orioles (Powell et al. 2014) used in our analyses. Node labels correspond to row labels in Supplement 7 Figures 3-6. Asterisks denote narrow-range Caribbean island endemics.

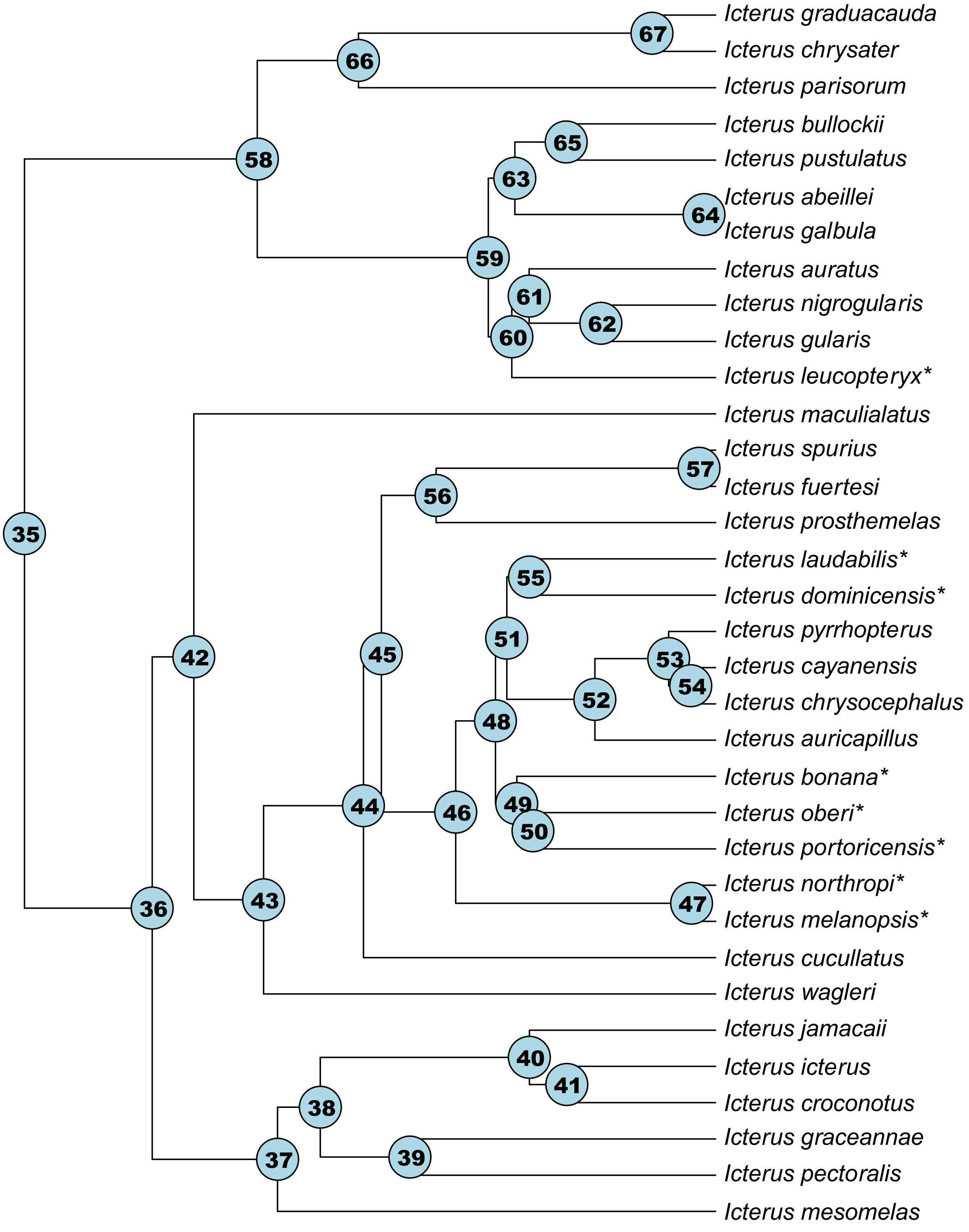

Supplementary Figure 2. Bin-based coding of annual mean temperature niche for New World Orioles (*Icterus* spp.). Phylogenetic relationships (Supplemental Figure 1) plotted on left side of figure. Dark red cells in a given row indicate bins that are inferred to be part of a species' fundamental niche (1). Pale red cells contain values at the unknown periphery of a species' fundamental niche (?). White cells are inferred to be values outside a species' fundamental niche (0).

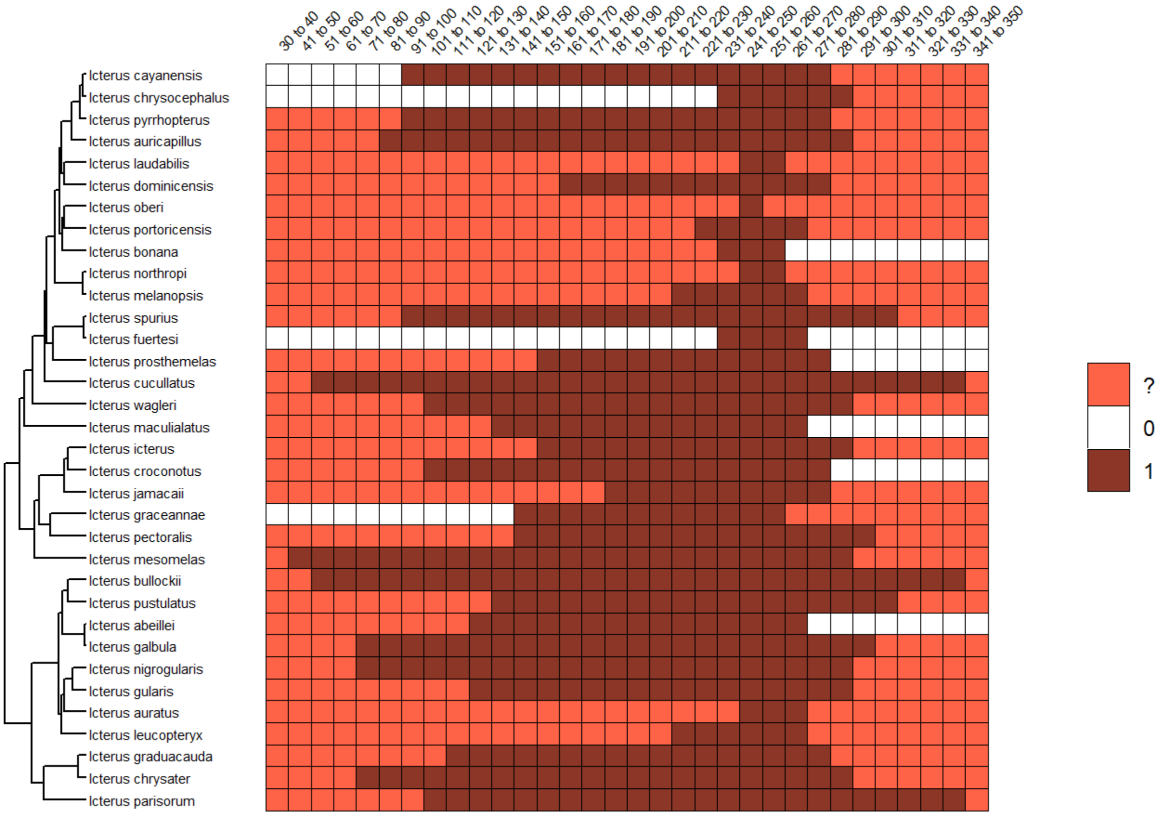

Supplementary Figure 3. Bin-based coding of annual precipitation niche for New World Orioles (*Icterus* spp.). Phylogenetic relationships (Supplemental Figure 1) plotted on left side of figure. Dark blue cells in a given row indicate bins that are inferred to be part of a species' fundamental niche (1). Pale blue cells contain values at the unknown periphery of a species' fundamental niche (?). White cells are inferred to be values outside a species' fundamental niche (0).

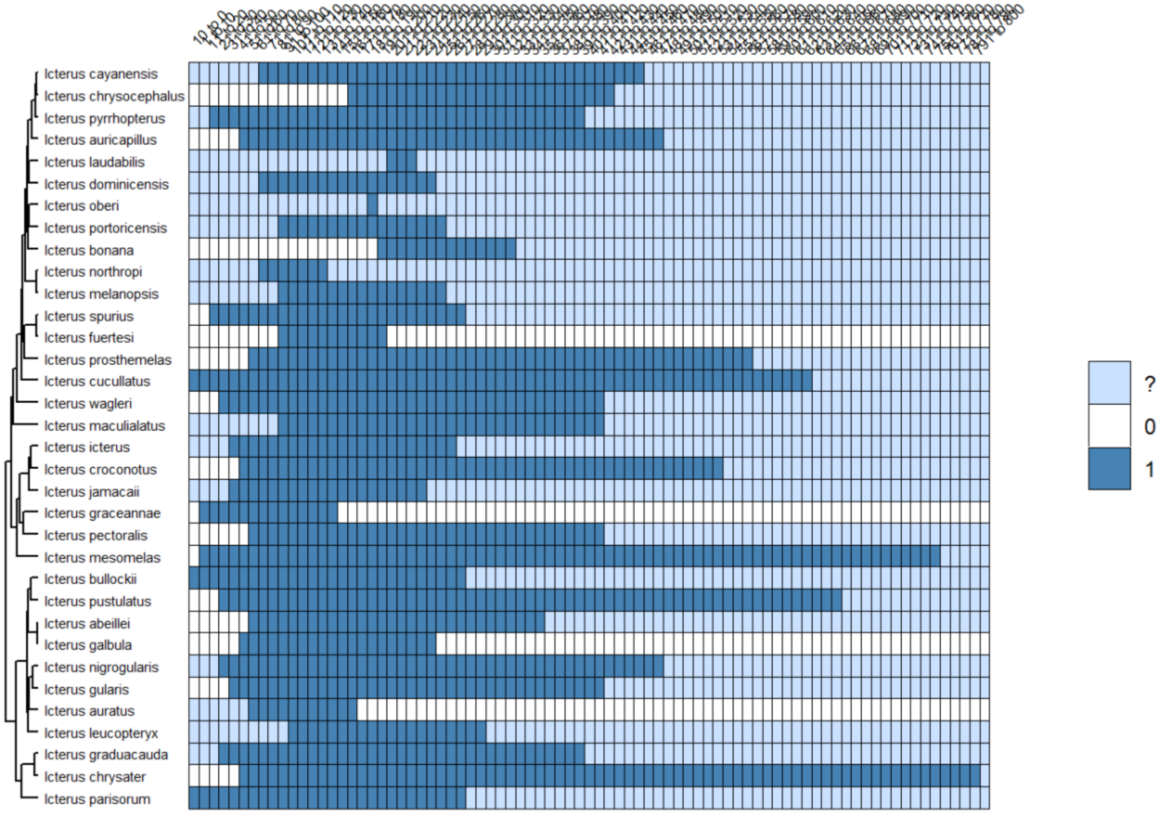

Supplementary Table 1. Summary of New World oriole (*Icterus* spp.) occurrence dataused in ecological niche characterizations. For migratory taxa (marked with an "x"), "Number" reflects species' breeding range occurrences.

Species	Migratory	Number
Icterus abeillei	x	572
Icterus auratus		1224
Icterus auricapillus		796
Icterus bonana		40
Icterus bullockii	x	57978
Icterus cayanensis		472
Icterus chrysater		4818
Icterus chrysocephalus		37
Icterus croconotus		1263
Icterus cucullatus	x	19458
Icterus dominicensis		509
Icterus fuertesi		38
Icterus galbula	x	180004
Icterus graceannae		485
Icterus graduacauda		2369
Icterus gularis		8130
Icterus icterus		1026
Icterus jamacaii		545
Icterus laudabilis		146
Icterus leucopteryx		743
Icterus maculialatus		318
Icterus melanopsis		771
Icterus mesomelas		2605
Icterus nigrogularis		3298
Icterus northropi		111
Icterus oberi		89
Icterus parisorum	x	6599
Icterus pectoralis		2085
Icterus portoricensis		1163

Supplementary Table 2. Maximum parsimony reconstruction table of New World oriole mean annual temperature niche. First column shows values obtained using median occurrence value; table cells bolded and boxed indicate which bin encompasses that value. Cell colors indicate bin score; "suitable" (1); dark gray, "unsuitable" (0): white; "unknown" (?): medium gray. Numbered rows indicate reconstructions of ancestral nodes corresponding to numbers in Supplementary Figure 1.

	Median from Occurrence	30 to 40	41 to 50	51 to 60	61 to 70	71 to 80	81 to 90	91 to 100	101 to 110	111 to 120	121 to 130	131 to 140	141 to 150	151 to 160	161 to 170	171 to 180	181 to 190	191 to 200	201 to 210	211 to 220	221 to 230	231 to 240	241 to 250	251 to 260	261 to 270	271 to 280	281 to 290	291 to 300	301 to 310	311 to 320	321 to 330	331 to 340	341 to 350
Icterus abeillei	170	?	?	?	?	?	?	?	?	?	1	1	1	1	**1**	1	1	1	1	1	1	1	1	1	1	0	0	0	0	0	0	0	0
Icterus bullockii	184	?	?	1	1	1	1	1	1	1	1	1	1	1	1	1	**1**	1	1	1	1	1	1	1	1	1	1	1	1	1	1	1	?
Icterus cucullatus	212	?	?	1	1	1	1	1	1	1	1	1	1	1	1	1	1	1	1	**1**	1	1	1	1	1	1	1	1	1	1	1	1	?
Icterus galbula	189	?	?	?	?	1	1	1	1	1	1	1	1	1	1	1	**1**	1	1	1	1	1	1	1	1	1	1	1	?	?	?	?	?
Icterus parisorum	228	?	?	?	?	?	?	?	1	1	1	1	1	1	1	1	1	1	1	1	**1**	1	1	1	1	1	1	1	1	1	1	1	?
Icterus pustulatus	263	?	?	?	?	?	?	?	?	?	?	1	1	1	1	1	1	1	1	1	1	1	1	1	**1**	1	1	1	1	?	?	?	?
Icterus spurius	213	?	?	?	?	?	?	1	1	1	1	1	1	1	1	1	1	1	1	**1**	1	1	1	1	1	1	1	1	1	?	?	?	?
Icterus auratus	257	?	?	?	?	?	?	?	?	?	?	?	?	?	?	?	?	?	?	?	?	?	1	**1**	1	?	?	?	?	?	?	?	?
Icterus auricapillus	254	?	?	?	?	?	1	1	1	1	1	1	1	1	1	1	1	1	1	1	1	1	1	**1**	1	1	1	?	?	?	?	?	?
Icterus bonana	240	?	?	?	?	?	?	?	?	?	?	?	?	?	?	?	?	?	?	?	?	**1**	1	1	0	0	0	0	0	0	0	0	0
Icterus cayanensis	255	0	0	0	0	0	0	1	1	1	1	1	1	1	1	1	1	1	1	1	1	1	1	**1**	1	1	?	?	?	?	?	?	?
Icterus chrysater	210	?	?	?	?	1	1	1	1	1	1	1	1	1	1	1	1	1	**1**	1	1	1	1	1	1	1	1	?	?	?	?	?	?
Icterus chrysocephalus	264	0	0	0	0	0	0	0	0	0	0	0	0	0	0	0	0	0	0	0	0	1	1	1	**1**	1	1	?	?	?	?	?	?
Icterus croconotus	254	?	?	?	?	?	?	?	1	1	1	1	1	1	1	1	1	1	1	1	1	1	1	**1**	1	1	?	?	?	?	?	?	?
Icterus dominicensis	252	?	?	?	?	?	?	?	?	?	?	?	?	?	1	1	1	1	1	1	1	1	1	**1**	1	1	?	?	?	?	?	?	?
Icterus fuertesi	242	0	0	0	0	0	0	0	0	0	0	0	0	0	0	0	0	0	0	0	0	1	**1**	1	1	0	0	0	0	0	0	0	0
Icterus graceannae	234	0	0	0	0	0	0	0	0	0	0	0	1	1	1	1	1	1	1	1	1	**1**	1	1	?	?	?	?	?	?	?	?	?
Icterus graduacauda	219	?	?	?	?	?	?	?	?	1	1	1	1	1	1	1	1	1	1	**1**	1	1	1	1	1	1	?	?	?	?	?	?	?
Icterus gularis	245	?	?	?	?	?	?	?	?	?	1	1	1	1	1	1	1	1	1	1	1	1	**1**	1	1	1	1	?	?	?	?	?	?
Icterus icterus	274	?	?	?	?	?	?	?	?	?	?	?	?	1	1	1	1	1	1	1	1	1	1	1	1	**1**	1	?	?	?	?	?	?
Icterus jamacaii	237	?	?	?	?	?	?	?	?	?	?	?	?	?	?	1	1	1	1	1	1	**1**	1	1	1	1	?	?	?	?	?	?	?
Icterus laudabilis	249	?	?	?	?	?	?	?	?	?	?	?	?	?	?	?	?	?	?	?	?	?	**1**	1	?	?	?	?	?	?	?	?	?
Icterus leucopteryx	241	?	?	?	?	?	?	?	?	?	?	?	?	?	?	?	?	?	?	1	1	1	**1**	1	1	?	?	?	?	?	?	?	?
Icterus maculialatus	221	?	?	?	?	?	?	?	?	?	?	1	1	1	1	1	1	1	1	1	**1**	1	1	1	1	0	0	0	0	0	0	0	0
Icterus melanopsis	251	?	?	?	?	?	?	?	?	?	?	?	?	?	?	?	?	?	?	1	1	1	**1**	1	1	?	?	?	?	?	?	?	?
Icterus mesomelas	252	?	1	1	1	1	1	1	1	1	1	1	1	1	1	1	1	1	1	1	1	1	1	**1**	1	1	1	?	?	?	?	?	?
Icterus nigrogularis	266	?	?	?	?	1	1	1	1	1	1	1	1	1	1	1	1	1	1	1	1	1	**1**	1	1	1	1	?	?	?	?	?	?
Icterus northropi	249	?	?	?	?	?	?	?	?	?	?	?	?	?	?	?	?	?	?	?	?	?	**1**	1	?	?	?	?	?	?	?	?	?
Icterus oberi	250	?	?	?	?	?	?	?	?	?	?	?	?	?	?	?	?	?	?	?	?	?	**1**	?	?	?	?	?	?	?	?	?	?
Icterus pectoralis	249	?	?	?	?	?	?	?	?	?	?	?	1	1	1	1	1	1	1	1	1	1	**1**	1	1	1	1	1	?	?	?	?	?
Icterus portoricensis	245	?	?	?	?	?	?	?	?	?	?	?	?	?	?	?	?	?	?	?	1	1	**1**	1	1	?	?	?	?	?	?	?	?
Icterus prosthemelas	252	?	?	?	?	?	?	?	?	?	?	?	?	1	1	1	1	1	1	1	1	**1**	1	1	1	1	0	0	0	0	0	0	0
Icterus pyrrhopterus	206	?	?	?	?	?	?	1	1	1	1	1	1	1	1	1	1	1	**1**	1	1	1	1	1	1	1	?	?	?	?	?	?	?
Icterus wagleri	196	?	?	?	?	?	?	?	1	1	1	1	1	1	1	1	1	1	**1**	1	1	1	1	1	1	1	1	?	?	?	?	?	?
35	228	0	0	0	0	0	0	0	0	0	0	0	0	0	0	0	0	0	0	0	**0**	1	1	1	1	1	1	1	1	1	1	1	1
36	226	0	0	0	0	0	0	0	0	0	0	0	0	0	0	0	0	0	0	0	**0**	1	1	1	1	1	1	1	1	1	1	1	1
37	212	0	0	0	0	0	0	0	0	0	0	0	0	0	0	0	0	0	0	**0**	0	1	1	1	1	1	1	1	1	1	1	1	1
38	212	0	0	0	0	0	0	0	0	0	0	0	0	0	0	0	0	0	0	**0**	0	1	1	1	1	1	1	1	1	1	1	1	0
39	206	0	0	0	0	0	0	0	0	0	0	0	0	0	0	0	0	0	**0**	0	0	1	1	1	1	1	1	1	1	1	1	1	0
40	225	0	0	0	0	0	0	0	0	0	0	0	0	0	0	0	0	0	0	0	**0**	1	1	1	1	1	1	1	1	1	1	1	0
41	219	0	0	0	0	0	0	0	0	0	0	0	0	0	0	0	0	0	0	**0**	0	1	1	1	1	1	1	1	1	1	1	1	0
42	229	0	0	0	0	0	0	0	0	0	0	0	0	0	0	0	0	0	0	0	**0**	1	1	1	1	1	1	1	1	1	1	1	1
43	233	0	0	0	0	0	0	0	0	0	0	0	0	0	0	0	0	0	0	0	0	**1**	1	1	1	1	1	1	1	1	1	1	1
44	244	0	0	0	0	0	0	0	0	0	0	0	0	0	0	0	0	0	0	0	0	1	**1**	1	1	1	1	1	1	1	1	1	1
45	245	0	0	0	0	0	0	0	0	0	0	0	0	0	0	0	0	0	0	0	0	1	**1**	1	1	1	1	1	1	1	1	1	0
46	244	0	0	0	0	0	0	0	0	0	0	0	0	0	0	0	0	0	0	0	0	1	**1**	1	1	1	1	1	1	1	1	1	0
47	247	0	0	0	0	0	0	0	0	0	0	0	0	0	0	0	0	0	0	0	0	1	**1**	1	1	1	1	1	1	1	1	1	0
48	243	0	0	0	0	0	0	0	0	0	0	0	0	0	0	0	0	0	0	0	0	1	**1**	1	1	1	1	1	1	1	1	1	0
49	243	0	?	?	?	?	?	?	?	?	?	?	?	?	?	?	?	?	?	?	?	1	**1**	1	1	1	1	1	1	1	1	1	0
50	242	0	?	?	?	?	?	?	?	?	?	?	?	?	?	?	?	?	?	?	?	1	**1**	1	1	1	1	1	1	1	1	1	0
51	242	0	0	0	0	0	0	0	0	0	0	0	0	0	0	0	0	0	0	0	0	1	**1**	1	1	1	1	1	1	1	1	1	0
52	246	0	0	0	0	0	0	0	0	0	0	0	0	0	0	0	0	0	0	0	0	1	**1**	1	1	1	1	1	1	1	1	1	0
53	242	0	0	0	0	0	0	0	0	0	0	0	0	0	0	0	0	0	0	0	0	1	**1**	1	1	1	1	1	1	1	1	1	0
54	245	?	1	1	1	1	1	1	1	1	1	1	1	1	1	1	1	1	1	1	1	1	**1**	1	1	1	1	1	1	1	1	1	0
55	240	0	0	0	0	0	0	0	0	0	0	0	0	0	0	0	0	0	0	0	0	**1**	1	1	1	1	1	1	1	1	1	1	0
56	249	0	0	0	0	0	0	0	0	0	0	0	0	0	0	0	0	0	0	0	0	1	**1**	1	0	0	0	0	0	0	0	0	0
57	243	0	0	0	0	0	0	0	0	0	0	0	0	0	0	0	0	0	0	0	0	1	**1**	1	0	0	0	0	0	0	0	0	0
58	234	0	0	0	0	0	0	0	0	0	0	0	0	0	0	0	0	0	0	0	0	**1**	1	1	1	1	1	1	1	1	1	1	1
59	246	0	0	0	0	0	0	0	0	0	0	0	0	0	0	0	0	0	0	0	0	1	**1**	1	1	1	1	1	1	1	1	1	1
60	246	0	0	0	0	0	0	0	0	0	0	0	0	0	0	0	0	0	0	0	0	1	**1**	1	1	1	1	1	1	1	1	1	0
61	248	0	0	0	0	0	0	0	0	0	0	0	0	0	0	0	0	0	0	0	0	1	**1**	1	1	1	1	1	1	1	1	1	0
62	250	0	0	0	0	0	0	0	0	0	0	0	0	0	0	0	0	0	0	0	0	1	**1**	1	1	1	1	1	1	1	1	1	1
63	246	0	0	0	0	0	0	0	0	0	0	0	0	0	0	0	0	0	0	0	0	1	**1**	1	1	1	1	1	1	1	1	1	1
64	249	0	0	0	0	0	0	0	0	0	0	0	0	0	0	0	0	0	0	0	0	1	**1**	1	1	1	1	1	1	1	1	1	1
65	247	0	0	0	0	0	0	0	0	0	0	0	0	0	0	0	0	0	0	0	0	1	**1**	1	1	1	1	1	1	1	1	1	1
66	231	0	0	0	0	0	0	0	0	0	0	0	0	0	0	0	0	0	0	0	0	**1**	1	1	1	1	1	1	1	1	1	1	1
67	204	0	0	0	0	0	0	0	0	0	0	0	0	0	0	0	0	0	**0**	0	0	1	1	1	1	1	1	1	1	1	1	1	1

Supplementary Table 3. Maximum likelihood reconstruction table of New World oriole mean annual temperature niche. First column shows values obtained using median occurrence value; table cells bolded and boxed indicate which bin encompasses that value. Cell colors indicate bin score; "suitable" (1); dark gray, "unsuitable" (0): white; "unknown" (?): medium gray. Numbered rows indicate reconstructions of ancestral nodes corresponding to numbers in Supplementary Figure 1.

	Median from Occurrence	30 to 40	41 to 50	51 to 60	61 to 70	71 to 80	81 to 90	91 to 100	101 to 110	111 to 120	121 to 130	131 to 140	141 to 150	151 to 160	161 to 170	171 to 180	181 to 190	191 to 200	201 to 210	211 to 220	221 to 230	231 to 240	241 to 250	251 to 260	261 to 270	271 to 280	281 to 290	291 to 300	301 to 310	311 to 320	321 to 330	331 to 340	341 to 350
Icterus abeillei	170	?	?	?	?	?	?	?	?	?	1	1	1	1	**1**	1	1	1	1	1	1	1	1	1	1	0	0	0	0	0	0	0	0
Icterus bullockii	184	?	?	1	1	1	1	1	1	1	1	1	1	1	1	1	**1**	1	1	1	1	1	1	1	1	1	1	1	1	1	1	1	?
Icterus cucullatus	212	?	?	1	1	1	1	1	1	1	1	1	1	1	1	1	1	1	1	**1**	1	1	1	1	1	1	1	1	1	1	1	1	?
Icterus galbula	189	?	?	?	?	1	1	1	1	1	1	1	1	1	1	1	**1**	1	1	1	1	1	1	1	1	1	1	1	?	?	?	?	?
Icterus parisorum	228	?	?	?	?	?	?	?	1	1	1	1	1	1	1	1	1	1	1	1	**1**	1	1	1	1	1	1	1	1	1	1	1	?
Icterus pustulatus	263	?	?	?	?	?	?	?	?	?	?	1	1	1	1	1	1	1	1	1	1	1	1	1	**1**	1	1	1	1	?	?	?	?
Icterus spurius	213	?	?	?	?	?	?	1	1	1	1	1	1	1	1	1	1	1	1	**1**	1	1	1	1	1	1	1	1	1	?	?	?	?
Icterus auratus	257	?	?	?	?	?	?	?	?	?	?	?	?	?	?	?	?	?	?	?	?	?	1	**1**	1	?	?	?	?	?	?	?	?
Icterus auricapillus	254	?	?	?	?	?	1	1	1	1	1	1	1	1	1	1	1	1	1	1	1	1	1	**1**	1	1	1	?	?	?	?	?	?
Icterus bonana	240	?	?	?	?	?	?	?	?	?	?	?	?	?	?	?	?	?	?	?	?	**1**	1	1	0	0	0	0	0	0	0	0	0
Icterus cayanensis	255	0	0	0	0	0	0	1	1	1	1	1	1	1	1	1	1	1	1	1	1	1	1	**1**	1	1	?	?	?	?	?	?	?
Icterus chrysater	210	?	?	?	?	1	1	1	1	1	1	1	1	1	1	1	1	1	**1**	1	1	1	1	1	1	1	1	?	?	?	?	?	?
Icterus chrysocephalus	264	0	0	0	0	0	0	0	0	0	0	0	0	0	0	0	0	0	0	0	0	1	1	1	**1**	1	1	?	?	?	?	?	?
Icterus croconotus	254	?	?	?	?	?	?	?	1	1	1	1	1	1	1	1	1	1	1	1	1	1	1	**1**	1	1	?	?	?	?	?	?	?
Icterus dominicensis	252	?	?	?	?	?	?	?	?	?	?	?	?	?	1	1	1	1	1	1	1	1	1	**1**	1	1	?	?	?	?	?	?	?
Icterus fuertesi	242	0	0	0	0	0	0	0	0	0	0	0	0	0	0	0	0	0	0	0	0	1	**1**	1	1	0	0	0	0	0	0	0	0
Icterus graceannae	234	0	0	0	0	0	0	0	0	0	0	0	1	1	1	1	1	1	1	1	1	**1**	1	1	?	?	?	?	?	?	?	?	?
Icterus graduacauda	219	?	?	?	?	?	?	?	?	1	1	1	1	1	1	1	1	1	1	**1**	1	1	1	1	1	1	?	?	?	?	?	?	?
Icterus gularis	245	?	?	?	?	?	?	?	?	?	1	1	1	1	1	1	1	1	1	1	1	1	**1**	1	1	1	1	?	?	?	?	?	?
Icterus icterus	274	?	?	?	?	?	?	?	?	?	?	?	?	1	1	1	1	1	1	1	1	1	1	1	1	**1**	1	?	?	?	?	?	?
Icterus jamacaii	237	?	?	?	?	?	?	?	?	?	?	?	?	?	?	1	1	1	1	1	1	**1**	1	1	1	1	?	?	?	?	?	?	?
Icterus laudabilis	249	?	?	?	?	?	?	?	?	?	?	?	?	?	?	?	?	?	?	?	?	?	**1**	1	?	?	?	?	?	?	?	?	?
Icterus leucopteryx	241	?	?	?	?	?	?	?	?	?	?	?	?	?	?	?	?	?	?	1	1	1	**1**	1	1	?	?	?	?	?	?	?	?
Icterus maculialatus	221	?	?	?	?	?	?	?	?	?	?	1	1	1	1	1	1	1	1	1	**1**	1	1	1	1	0	0	0	0	0	0	0	0
Icterus melanopsis	251	?	?	?	?	?	?	?	?	?	?	?	?	?	?	?	?	?	?	1	1	1	**1**	1	1	?	?	?	?	?	?	?	?
Icterus mesomelas	252	?	1	1	1	1	1	1	1	1	1	1	1	1	1	1	1	1	1	1	1	1	1	**1**	1	1	1	?	?	?	?	?	?
Icterus nigrogularis	266	?	?	?	?	1	1	1	1	1	1	1	1	1	1	1	1	1	1	1	1	1	**1**	1	1	1	1	?	?	?	?	?	?
Icterus northropi	249	?	?	?	?	?	?	?	?	?	?	?	?	?	?	?	?	?	?	?	?	?	**1**	1	?	?	?	?	?	?	?	?	?
Icterus oberi	250	?	?	?	?	?	?	?	?	?	?	?	?	?	?	?	?	?	?	?	?	?	**1**	?	?	?	?	?	?	?	?	?	?
Icterus pectoralis	249	?	?	?	?	?	?	?	?	?	?	?	1	1	1	1	1	1	1	1	1	1	**1**	1	1	1	1	1	?	?	?	?	?
Icterus portoricensis	245	?	?	?	?	?	?	?	?	?	?	?	?	?	?	?	?	?	?	?	1	1	**1**	1	1	?	?	?	?	?	?	?	?
Icterus prosthemelas	252	?	?	?	?	?	?	?	?	?	?	?	?	1	1	1	1	1	1	1	1	**1**	1	1	1	1	0	0	0	0	0	0	0
Icterus pyrrhopterus	206	?	?	?	?	?	?	1	1	1	1	1	1	1	1	1	1	1	**1**	1	1	1	1	1	1	1	?	?	?	?	?	?	?
Icterus wagleri	196	?	?	?	?	?	?	?	1	1	1	1	1	1	1	1	1	1	**1**	1	1	1	1	1	1	1	1	?	?	?	?	?	?
35	228	?	?	?	?	?	?	?	?	?	?	1	1	1	1	1	1	1	1	1	**1**	1	1	1	1	1	?	?	?	?	?	?	?
36	226	?	?	?	?	?	?	?	?	?	?	1	1	1	1	1	1	1	1	1	**1**	1	1	1	1	1	?	?	?	?	?	?	?
37	212	?	?	?	?	?	?	?	?	?	?	?	1	1	1	1	1	1	1	**1**	1	1	1	1	1	1	?	?	?	?	?	?	?
38	212	?	?	?	?	?	?	?	?	?	?	?	1	1	1	1	1	1	1	**1**	1	1	1	1	1	1	?	?	?	?	?	?	?
39	206	?	?	?	?	?	?	?	?	?	?	?	1	1	1	1	1	1	**1**	1	1	1	1	1	1	1	?	?	?	?	?	?	?
40	225	?	?	?	?	?	?	?	?	?	?	?	?	1	1	1	1	1	1	1	**1**	1	1	1	1	1	?	?	?	?	?	?	?
41	219	?	?	?	?	?	?	?	?	?	?	?	?	1	1	1	1	1	1	**1**	1	1	1	1	1	1	?	?	?	?	?	?	?
42	229	?	?	?	?	?	?	?	?	?	?	1	1	1	1	1	1	1	1	1	**1**	1	1	1	1	1	?	?	?	?	?	?	?
43	233	?	?	?	?	?	?	?	?	?	?	1	1	1	1	1	1	1	1	1	1	**1**	1	1	1	1	?	?	?	?	?	?	?
44	244	?	?	?	?	?	?	?	?	?	?	?	1	1	1	1	1	1	1	1	1	1	**1**	1	1	1	?	?	?	?	?	?	?
45	245	?	?	?	?	?	?	?	?	?	?	?	?	1	1	1	1	1	1	1	1	1	**1**	1	1	1	?	?	?	?	?	?	?
46	244	?	?	?	?	?	?	?	?	?	?	?	?	?	?	?	?	?	?	1	1	1	**1**	1	1	1	?	?	?	?	?	?	?
47	247	?	?	?	?	?	?	?	?	?	?	?	?	?	?	?	?	?	?	1	1	1	**1**	1	1	?	?	?	?	?	?	?	?
48	243	?	?	?	?	?	?	?	?	?	?	?	?	?	?	?	?	?	?	1	1	1	**1**	1	1	1	?	?	?	?	?	?	?
49	243	?	?	?	?	?	?	?	?	?	?	?	?	?	?	?	?	?	?	?	1	1	**1**	1	1	?	?	?	?	?	?	?	?
50	242	?	?	?	?	?	?	?	?	?	?	?	?	?	?	?	?	?	?	?	1	1	**1**	1	1	?	?	?	?	?	?	?	?
51	242	?	?	?	?	?	?	?	?	?	?	?	?	?	?	?	?	?	?	1	1	1	**1**	1	1	1	?	?	?	?	?	?	?
52	246	?	?	?	?	?	?	1	1	1	1	1	1	1	1	1	1	1	1	1	1	1	**1**	1	1	1	?	?	?	?	?	?	?
53	242	?	?	?	?	?	?	1	1	1	1	1	1	1	1	1	1	1	1	1	1	1	**1**	1	1	1	?	?	?	?	?	?	?
54	245	0	0	0	0	0	0	1	1	1	1	1	1	1	1	1	1	1	1	1	1	1	**1**	1	1	1	?	?	?	?	?	?	?
55	240	?	?	?	?	?	?	?	?	?	?	?	?	?	?	?	?	?	?	1	1	**1**	1	1	1	1	?	?	?	?	?	?	?
56	249	?	?	?	?	?	?	?	?	?	?	?	?	1	1	1	1	1	1	1	1	1	**1**	1	1	1	?	?	?	?	?	?	?
57	243	?	?	?	?	?	?	1	1	1	1	1	1	1	1	1	1	1	1	1	1	1	**1**	1	1	1	?	?	?	?	?	?	?
58	234	?	?	?	?	?	?	?	?	?	1	1	1	1	1	1	1	1	1	1	1	**1**	1	1	1	1	?	?	?	?	?	?	?
59	246	?	?	?	?	?	?	?	?	?	1	1	1	1	1	1	1	1	1	1	1	1	**1**	1	1	1	?	?	?	?	?	?	?
60	246	?	?	?	?	?	?	?	?	?	1	1	1	1	1	1	1	1	1	1	1	1	**1**	1	1	1	?	?	?	?	?	?	?
61	248	?	?	?	?	?	?	?	?	?	1	1	1	1	1	1	1	1	1	1	1	1	**1**	1	1	1	?	?	?	?	?	?	?
62	250	?	?	?	?	?	?	?	?	?	1	1	1	1	1	1	1	1	1	1	1	1	**1**	1	1	1	?	?	?	?	?	?	?
63	246	?	?	?	?	?	?	?	?	?	1	1	1	1	1	1	1	1	1	1	1	1	**1**	1	1	1	1	1	?	?	?	?	?
64	249	?	?	?	?	?	?	?	?	?	1	1	1	1	1	1	1	1	1	1	1	1	**1**	1	1	1	1	1	?	?	?	?	?
65	247	?	?	?	?	?	?	?	?	?	1	1	1	1	1	1	1	1	1	1	1	1	**1**	1	1	1	1	1	1	?	?	?	?
66	231	?	?	?	?	?	?	?	?	?	1	1	1	1	1	1	1	1	1	1	1	**1**	1	1	1	1	?	?	?	?	?	?	?
67	204	?	?	?	?	?	?	?	?	?	1	1	1	1	1	1	1	1	**1**	1	1	1	1	1	1	1	?	?	?	?	?	?	?

Supplementary Table 4. Maximum parsimony reconstruction table of New World oriole annual precipitation niche. First column shows values obtained using median occurrence value; table cells bolded and boxed indicate which bin encompasses that value. Cell colors indicate bin score; “suitable” (1); dark gray, “unsuitable” (0): white; “unknown” (?): medium gray. Numbered rows indicate reconstructions of ancestral nodes corresponding to numbers in Supplementary Figure 1.

	Median from Occurrence	-10 to 0	1 to 10	11 to 20	21 to 30	31 to 40	41 to 50	51 to 60	61 to 70	71 to 80	81 to 90	91 to 100	101 to 110	111 to 120	121 to 130	131 to 140	141 to 150	151 to 160	161 to 170	171 to 180	181 to 190	191 to 200	201 to 210	211 to 220	221 to 230	231 to 240	241 to 250	251 to 260	261 to 270	271 to 280	281 to 290	291 to 300	301 to 310	311 to 320	321 to 330	331 to 340	341 to 350	351 to 360	361 to 370	371 to 380	381 to 390	391 to 400	401 to 410	411 to 420	421 to 430	431 to 440	441 to 450	451 to 460	461 to 470	471 to 480	481 to 490	491 to 500	501 to 510	511 to 520	521 to 530	531 to 540	541 to 550	551 to 560	561 to 570	571 to 580	581 to 590	591 to 600	601 to 610	611 to 620	621 to 630	631 to 640	641 to 650	651 to 660	661 to 670	671 to 680	681 to 690	691 to 700	701 to 710	711 to 720	721 to 730	731 to 740	741 to 750	751 to 760	761 to 770	771 to 780	781 to 790	791 to 800
Icterus abeillei	166	0	0	0	0	0	0	1	1	1	1	1	1	1	1	1	1	1	**1**	1	1	1	1	1	1	1	1	1	1	1	1	1	1	1	1	1	1	?	?	?	?	?	?	?	?	?	?	?	?	?	?	?	?	?	?	?	?	?	?	?	?	?	?	?	?	?	?	?	?	?	?	?	?	?	?	?	?	?	?	?	?	?	?	?
Icterus bullockii	32	1	1	1	1	**1**	1	1	1	1	1	1	1	1	1	1	1	1	1	1	1	1	1	1	1	1	1	1	1	?	?	?	?	?	?	?	?	?	?	?	?	?	?	?	?	?	?	?	?	?	?	?	?	?	?	?	?	?	?	?	?	?	?	?	?	?	?	?	?	?	?	?	?	?	?	?	?	?	?	?	?	?	?	?
Icterus cucullatus	6	1	**1**	1	1	1	1	1	1	1	1	1	1	1	1	1	1	1	1	1	1	1	1	1	1	1	1	1	1	1	1	1	1	1	1	1	1	1	1	1	1	1	1	1	1	1	1	1	1	1	1	1	1	1	1	1	1	1	1	1	1	1	1	1	?	?	?	?	?	?	?	?	?	?	?	?	?	?	?	?	?	?
Icterus galbula	113	0	0	0	0	0	1	1	1	1	1	1	1	**1**	1	1	1	1	1	1	1	1	1	1	1	1	0	0	0	0	0	0	0	0	0	0	0	0	0	0	0	0	0	0	0	0	0	0	0	0	0	0	0	0	0	0	0	0	0	0	0	0	0	0	0	0	0	0	0	0	0	0	0	0	0	0	0	0	0	0	0	0
Icterus parisorum	50	1	1	1	1	1	**1**	1	1	1	1	1	1	1	1	1	1	1	1	1	1	1	1	1	1	1	1	1	1	?	?	?	?	?	?	?	?	?	?	?	?	?	?	?	?	?	?	?	?	?	?	?	?	?	?	?	?	?	?	?	?	?	?	?	?	?	?	?	?	?	?	?	?	?	?	?	?	?	?	?	?	?	?	?
Icterus pustulatus	200	0	0	0	1	1	1	1	1	1	1	1	1	1	1	1	1	1	1	1	1	**1**	1	1	1	1	1	1	1	1	1	1	1	1	1	1	1	1	1	1	1	1	1	1	1	1	1	1	1	1	1	1	1	1	1	1	1	1	1	1	1	1	1	1	1	1	1	?	?	?	?	?	?	?	?	?	?	?	?	?	?	?
Icterus spurius	119	0	0	1	1	1	1	1	1	1	1	1	1	1	1	1	1	1	1	1	**1**	1	1	1	1	1	1	1	1	?	?	?	?	?	?	?	?	?	?	?	?	?	?	?	?	?	?	?	?	?	?	?	?	?	?	?	?	?	?	?	?	?	?	?	?	?	?	?	?	?	?	?	?	?	?	?	?	?	?	?	?	?	?	?
Icterus auratus	113	?	?	?	?	?	?	1	1	1	1	1	1	**1**	1	1	1	1	0	0	0	0	0	0	0	0	0	0	0	0	0	0	0	0	0	0	0	0	0	0	0	0	0	0	0	0	0	0	0	0	0	0	0	0	0	0	0	0	0	0	0	0	0	0	0	0	0	0	0	0	0	0	0	0	0	0	0	0	0	0	0	0
Icterus auricapillus	183	0	0	0	0	0	1	1	1	1	1	1	1	1	1	1	1	1	1	1	**1**	1	1	1	1	1	1	1	1	1	1	1	1	1	1	1	1	1	1	1	1	1	1	1	1	1	1	1	1	?	?	?	?	?	?	?	?	?	?	?	?	?	?	?	?	?	?	?	?	?	?	?	?	?	?	?	?	?	?	?	?	?	?	?
Icterus bonana	274	0	0	0	0	0	0	0	0	0	0	0	0	0	0	0	0	0	0	0	1	1	1	1	1	1	1	1	1	**1**	1	1	1	1	?	?	?	?	?	?	?	?	?	?	?	?	?	?	?	?	?	?	?	?	?	?	?	?	?	?	?	?	?	?	?	?	?	?	?	?	?	?	?	?	?	?	?	?	?	?	?	?	?
Icterus cayanensis	236	?	?	?	?	?	?	?	1	1	1	1	1	1	1	1	1	1	1	1	1	1	1	1	1	**1**	1	1	1	1	1	1	1	1	1	1	1	1	1	1	1	1	1	1	1	1	1	?	?	?	?	?	?	?	?	?	?	?	?	?	?	?	?	?	?	?	?	?	?	?	?	?	?	?	?	?	?	?	?	?	?	?	?	?
Icterus chrysater	173	0	0	0	0	0	1	1	1	1	1	1	1	1	1	1	1	1	1	**1**	1	1	1	1	1	1	1	1	1	1	1	1	1	1	1	1	1	1	1	1	1	1	1	1	1	1	1	1	1	1	1	1	1	1	1	1	1	1	1	1	1	1	1	1	1	1	1	1	1	1	1	1	1	1	1	1	1	1	1	1	1	?
Icterus chrysocephalus	263	0	0	0	0	0	0	0	0	0	0	0	0	0	0	0	0	1	1	1	1	1	1	1	1	1	1	1	**1**	1	1	1	1	1	1	1	1	1	1	1	1	1	1	1	?	?	?	?	?	?	?	?	?	?	?	?	?	?	?	?	?	?	?	?	?	?	?	?	?	?	?	?	?	?	?	?	?	?	?	?	?	?	?	?
Icterus croconotus	168	0	0	0	0	0	1	1	1	1	1	1	1	1	1	1	1	1	**1**	1	1	1	1	1	1	1	1	1	1	1	1	1	1	1	1	1	1	1	1	1	1	1	1	1	1	1	1	1	1	1	1	1	1	1	1	?	?	?	?	?	?	?	?	?	?	?	?	?	?	?	?	?	?	?	?	?	?	?	?	?	?	?	?	?
Icterus dominicensis	130	?	?	?	?	?	?	?	1	1	1	1	1	1	**1**	1	1	1	1	1	1	1	1	1	1	1	?	?	?	?	?	?	?	?	?	?	?	?	?	?	?	?	?	?	?	?	?	?	?	?	?	?	?	?	?	?	?	?	?	?	?	?	?	?	?	?	?	?	?	?	?	?	?	?	?	?	?	?	?	?	?	?	?	?
Icterus fuertesi	114	0	0	0	0	0	0	0	0	0	1	1	1	**1**	1	1	1	1	1	1	1	0	0	0	0	0	0	0	0	0	0	0	0	0	0	0	0	0	0	0	0	0	0	0	0	0	0	0	0	0	0	0	0	0	0	0	0	0	0	0	0	0	0	0	0	0	0	0	0	0	0	0	0	0	0	0	0	0	0	0	0	0
Icterus graceannae	29	0	1	1	**1**	1	1	1	1	1	1	1	1	1	1	1	0	0	0	0	0	0	0	0	0	0	0	0	0	0	0	0	0	0	0	0	0	0	0	0	0	0	0	0	0	0	0	0	0	0	0	0	0	0	0	0	0	0	0	0	0	0	0	0	0	0	0	0	0	0	0	0	0	0	0	0	0	0	0	0	0	0
Icterus graduacauda	71	?	?	?	1	1	1	1	1	**1**	1	1	1	1	1	1	1	1	1	1	1	1	1	1	1	1	1	1	1	1	1	1	1	1	1	1	1	1	1	1	1	?	?	?	?	?	?	?	?	?	?	?	?	?	?	?	?	?	?	?	?	?	?	?	?	?	?	?	?	?	?	?	?	?	?	?	?	?	?	?	?	?	?	?
Icterus gularis	117	0	0	0	0	1	1	1	1	1	1	1	1	**1**	1	1	1	1	1	1	1	1	1	1	1	1	1	1	1	1	1	1	1	1	1	1	1	1	1	1	1	1	1	?	?	?	?	?	?	?	?	?	?	?	?	?	?	?	?	?	?	?	?	?	?	?	?	?	?	?	?	?	?	?	?	?	?	?	?	?	?	?	?	?
Icterus icterus	48	?	?	?	?	1	**1**	1	1	1	1	1	1	1	1	1	1	1	1	1	1	1	1	1	1	1	1	1	1	1	1	1	?	?	?	?	?	?	?	?	?	?	?	?	?	?	?	?	?	?	?	?	?	?	?	?	?	?	?	?	?	?	?	?	?	?	?	?	?	?	?	?	?	?	?	?	?	?	?	?	?	?	?	?
Icterus jamacaii	108	?	?	?	?	1	1	1	1	1	1	1	**1**	1	1	1	1	1	1	1	1	1	1	1	1	?	?	?	?	?	?	?	?	?	?	?	?	?	?	?	?	?	?	?	?	?	?	?	?	?	?	?	?	?	?	?	?	?	?	?	?	?	?	?	?	?	?	?	?	?	?	?	?	?	?	?	?	?	?	?	?	?	?	?
Icterus laudabilis	205	?	?	?	?	?	?	?	?	?	?	?	?	?	?	?	?	?	?	?	?	1	**1**	1	?	?	?	?	?	?	?	?	?	?	?	?	?	?	?	?	?	?	?	?	?	?	?	?	?	?	?	?	?	?	?	?	?	?	?	?	?	?	?	?	?	?	?	?	?	?	?	?	?	?	?	?	?	?	?	?	?	?	?	?
Icterus leucopteryx	200	?	?	?	?	?	?	?	?	?	?	1	1	1	1	1	1	1	1	1	1	1	**1**	1	1	1	1	1	1	1	1	?	?	?	?	?	?	?	?	?	?	?	?	?	?	?	?	?	?	?	?	?	?	?	?	?	?	?	?	?	?	?	?	?	?	?	?	?	?	?	?	?	?	?	?	?	?	?	?	?	?	?	?	?
Icterus maculialatus	136	?	?	?	?	?	?	?	?	?	1	1	1	1	1	**1**	1	1	1	1	1	1	1	1	1	1	1	1	1	1	1	1	1	1	1	1	1	1	1	1	1	1	1	?	?	?	?	?	?	?	?	?	?	?	?	?	?	?	?	?	?	?	?	?	?	?	?	?	?	?	?	?	?	?	?	?	?	?	?	?	?	?	?	?
Icterus melanopsis	133	?	?	?	?	?	?	?	?	?	1	1	1	1	1	**1**	1	1	1	1	1	1	1	1	1	1	1	?	?	?	?	?	?	?	?	?	?	?	?	?	?	?	?	?	?	?	?	?	?	?	?	?	?	?	?	?	?	?	?	?	?	?	?	?	?	?	?	?	?	?	?	?	?	?	?	?	?	?	?	?	?	?	?	?
Icterus mesomelas	179	0	1	1	1	1	1	1	1	1	1	1	1	1	1	1	1	1	1	**1**	1	1	1	1	1	1	1	1	1	1	1	1	1	1	1	1	1	1	1	1	1	1	1	1	1	1	1	1	1	1	1	1	1	1	1	1	1	1	1	1	1	1	1	1	1	1	1	1	1	1	1	1	1	1	1	1	1	?	?	?	?	?
Icterus nigrogularis	135	?	?	?	1	1	1	1	1	1	1	1	1	1	1	**1**	1	1	1	1	1	1	1	1	1	1	1	1	1	1	1	1	1	1	1	1	1	1	1	1	1	1	1	1	1	1	1	1	1	?	?	?	?	?	?	?	?	?	?	?	?	?	?	?	?	?	?	?	?	?	?	?	?	?	?	?	?	?	?	?	?	?	?	?
Icterus northropi	124	?	?	?	?	?	?	?	1	1	1	1	1	1	**1**	?	?	?	?	?	?	?	?	?	?	?	?	?	?	?	?	?	?	?	?	?	?	?	?	?	?	?	?	?	?	?	?	?	?	?	?	?	?	?	?	?	?	?	?	?	?	?	?	?	?	?	?	?	?	?	?	?	?	?	?	?	?	?	?	?	?	?	?	?
Icterus oberi	178	?	?	?	?	?	?	?	?	?	?	?	?	?	?	?	?	?	?	**1**	?	?	?	?	?	?	?	?	?	?	?	?	?	?	?	?	?	?	?	?	?	?	?	?	?	?	?	?	?	?	?	?	?	?	?	?	?	?	?	?	?	?	?	?	?	?	?	?	?	?	?	?	?	?	?	?	?	?	?	?	?	?	?	?
Icterus pectoralis	162	0	0	0	0	0	0	1	1	1	1	1	1	1	1	1	1	1	**1**	1	1	1	1	1	1	1	1	1	1	1	1	1	1	1	1	1	1	1	1	1	1	1	1	?	?	?	?	?	?	?	?	?	?	?	?	?	?	?	?	?	?	?	?	?	?	?	?	?	?	?	?	?	?	?	?	?	?	?	?	?	?	?	?	?
Icterus portoricensis	185	?	?	?	?	?	?	?	?	?	1	1	1	1	1	1	1	1	1	1	**1**	1	1	1	1	1	1	?	?	?	?	?	?	?	?	?	?	?	?	?	?	?	?	?	?	?	?	?	?	?	?	?	?	?	?	?	?	?	?	?	?	?	?	?	?	?	?	?	?	?	?	?	?	?	?	?	?	?	?	?	?	?	?	?
Icterus prosthemelas	235	0	0	0	0	0	0	1	1	1	1	1	1	1	1	1	1	1	1	1	1	1	1	1	1	**1**	1	1	1	1	1	1	1	1	1	1	1	1	1	1	1	1	1	1	1	1	1	1	1	1	1	1	1	1	1	1	1	1	?	?	?	?	?	?	?	?	?	?	?	?	?	?	?	?	?	?	?	?	?	?	?	?	?	?
Icterus pyrrhopterus	123	?	?	1	1	1	1	1	1	1	1	1	1	1	**1**	1	1	1	1	1	1	1	1	1	1	1	1	1	1	1	1	1	1	1	1	1	1	1	1	1	1	?	?	?	?	?	?	?	?	?	?	?	?	?	?	?	?	?	?	?	?	?	?	?	?	?	?	?	?	?	?	?	?	?	?	?	?	?	?	?	?	?	?	?
Icterus wagleri	100	0	0	0	1	1	1	1	1	1	1	**1**	1	1	1	1	1	1	1	1	1	1	1	1	1	1	1	1	1	1	1	1	1	1	1	1	1	1	1	1	1	1	1	?	?	?	?	?	?	?	?	?	?	?	?	?	?	?	?	?	?	?	?	?	?	?	?	?	?	?	?	?	?	?	?	?	?	?	?	?	?	?	?	?
35	141	0	?	?	?	?	?	?	?	?	?	?	?	?	?	?	**?**	?	?	?	?	?	?	?	?	?	?	?	?	?	?	?	?	?	?	?	?	?	?	?	?	?	?	?	?	?	?	?	?	?	?	?	?	?	?	?	?	?	?	?	?	?	?	?	?	?	?	?	?	?	?	?	?	?	?	?	?	0	0	0	0	0
36	133	0	?	?	?	?	?	?	?	?	?	?	?	?	?	**?**	?	?	?	?	?	?	?	?	?	?	?	?	?	?	?	?	?	?	?	?	?	?	?	?	?	?	?	?	?	?	?	?	?	?	?	?	?	?	?	?	?	?	?	?	?	?	?	?	?	?	?	?	?	?	?	?	?	?	?	?	?	0	0	0	0	0
37	112	0	?	?	?	?	?	?	?	?	?	?	?	**?**	?	?	?	?	?	?	?	?	?	?	?	?	?	?	?	?	?	?	?	?	?	?	?	?	?	?	?	?	?	?	?	?	?	?	?	?	?	?	?	?	?	?	?	?	?	?	?	?	?	?	?	?	?	?	?	?	?	?	?	?	?	?	?	0	0	0	0	0
38	101	0	?	?	?	?	?	?	?	?	?	?	**?**	?	?	?	?	?	?	?	?	?	?	?	?	?	?	?	?	?	?	?	?	?	?	?	?	?	?	?	?	?	?	?	?	?	?	?	?	?	?	?	?	?	?	?	?	?	?	?	?	?	?	?	?	?	?	?	?	?	?	?	?	?	?	?	?	0	0	0	0	0
39	69	0	?	?	?	?	?	?	**?**	?	?	?	?	?	?	?	?	?	?	?	?	?	?	?	?	?	?	?	?	?	?	?	?	?	?	?	?	?	?	?	?	?	?	?	?	?	?	?	?	?	?	?	?	?	?	?	?	?	?	?	?	?	?	?	?	?	?	?	?	?	?	?	?	?	?	?	?	0	0	0	0	0
40	119	?	?	?	?	?	?	?	?	?	?	?	?	**?**	?	?	?	?	?	?	?	?	?	?	?	?	?	?	?	?	?	?	?	?	?	?	?	?	?	?	?	?	?	?	?	?	?	?	?	?	?	?	?	?	?	?	?	?	?	?	?	?	?	?	?	?	?	?	?	?	?	?	?	?	?	?	?	0	0	0	0	0
41	106	?	?	?	?	?	?	?	?	?	?	?	**?**	?	?	?	?	?	?	?	?	?	?	?	?	?	?	?	?	?	?	?	?	?	?	?	?	?	?	?	?	?	?	?	?	?	?	?	?	?	?	?	?	?	?	?	?	?	?	?	?	?	?	?	?	?	?	?	?	?	?	?	?	?	?	?	?	0	0	0	0	0
42	137	0	?	?	?	?	?	?	?	?	?	?	?	?	?	**?**	?	?	?	?	?	?	?	?	?	?	?	?	?	?	?	?	?	?	?	?	?	?	?	?	?	?	?	?	?	?	?	?	?	?	?	?	?	?	?	?	?	?	?	?	?	?	?	?	?	?	?	?	?	?	?	?	?	?	?	?	?	0	0	0	0	0
43	136	0	1	1	?	?	?	?	?	?	?	?	?	?	?	**?**	?	?	?	?	?	?	?	?	?	?	?	?	?	?	?	?	?	?	?	?	?	?	?	?	?	?	?	?	?	?	?	?	?	?	?	?	?	?	?	?	?	?	?	?	?	?	?	?	?	?	?	?	?	?	?	?	?	?	?	?	?	0	0	0	0	0
44	138	0	1	1	?	?	?	?	?	?	?	?	?	?	?	**?**	?	?	?	?	?	?	?	?	?	?	?	?	?	?	?	?	?	?	?	?	?	?	?	?	?	?	?	?	?	?	?	?	?	?	?	?	?	?	?	?	?	?	?	?	?	?	?	?	?	?	?	?	?	?	?	?	?	?	?	?	?	0	0	0	0	0
45	139	0	1	1	?	?	?	?	?	?	?	?	?	?	?	**?**	?	?	?	?	?	?	?	?	?	?	?	?	?	?	?	?	?	?	?	?	?	?	?	?	?	?	?	?	?	?	?	?	?	?	?	?	?	?	?	?	?	?	?	?	?	?	?	?	?	?	?	?	?	?	?	?	?	?	?	?	?	0	0	0	0	0
46	160	?	?	?	?	?	?	?	?	?	?	?	?	?	?	?	?	**?**	?	?	?	?	?	?	?	?	?	?	?	?	?	?	?	?	?	?	?	?	?	?	?	?	?	?	?	?	?	?	?	?	?	?	?	?	?	?	?	?	?	?	?	?	?	?	?	?	?	?	?	?	?	?	?	?	?	?	?	0	0	0	0	0
47	225	?	?	?	?	?	?	?	?	?	?	?	?	?	?	?	?	?	?	?	?	?	?	?	**?**	?	?	?	?	?	?	?	?	?	?	?	?	?	?	?	?	?	?	?	?	?	?	?	?	?	?	?	?	?	?	?	?	?	?	?	?	?	?	?	?	?	?	?	?	?	?	?	?	?	?	?	?	0	0	0	0	0
48	160	?	?	?	?	?	?	?	?	?	?	?	?	?	?	?	?	**?**	?	?	?	?	?	?	?	?	?	?	?	?	?	?	?	?	?	?	?	?	?	?	?	?	?	?	?	?	?	?	?	?	?	?	?	?	?	?	?	?	?	?	?	?	?	?	?	?	?	?	?	?	?	?	?	?	?	?	?	0	0	0	0	0
49	176	?	?	?	?	?	?	?	?	?	?	?	?	?	?	?	?	?	?	**?**	?	?	?	?	?	?	?	?	?	?	?	?	?	?	?	?	?	?	?	?	?	?	?	?	?	?	?	?	?	?	?	?	?	?	?	?	?	?	?	?	?	?	?	?	?	?	?	?	?	?	?	?	?	?	?	?	?	0	0	0	0	0
50	180	?	?	?	?	?	?	?	?	?	?	?	?	?	?	?	?	?	?	**?**	?	?	?	?	?	?	?	?	?	?	?	?	?	?	?	?	?	?	?	?	?	?	?	?	?	?	?	?	?	?	?	?	?	?	?	?	?	?	?	?	?	?	?	?	?	?	?	?	?	?	?	?	?	?	?	?	?	0	0	0	0	0
51	152	?	?	?	?	?	?	?	?	?	?	?	?	?	?	?	?	**?**	?	?	?	?	?	?	?	?	?	?	?	?	?	?	?	?	?	?	?	?	?	?	?	?	?	?	?	?	?	?	?	?	?	?	?	?	?	?	?	?	?	?	?	?	?	?	?	?	?	?	?	?	?	?	?	?	?	?	?	0	0	0	0	0
52	132	0	0	1	1	1	?	?	?	?	?	?	?	?	?	**?**	?	?	?	?	?	?	?	?	?	?	?	?	?	?	?	?	?	?	?	?	?	?	?	?	?	?	?	?	?	?	?	?	?	?	?	?	?	?	?	?	?	?	?	?	?	?	?	?	?	?	?	?	?	?	?	?	?	?	?	?	?	0	0	0	0	0
53	93	0	0	1	1	1	?	?	?	?	?	**?**	?	?	?	?	?	?	?	?	?	?	?	?	?	?	?	?	?	?	?	?	?	?	?	?	?	?	?	?	?	?	?	?	?	?	?	?	?	?	?	?	?	?	?	?	?	?	?	?	?	?	?	?	?	?	?	?	?	?	?	?	?	?	?	?	?	0	0	0	0	0
54	111	0	0	1	1	1	0	0	0	0	0	0	0	**0**	0	0	0	0	0	0	0	0	0	0	0	0	0	0	0	0	0	0	0	0	0	0	0	0	0	0	0	0	0	0	0	0	0	0	0	0	0	0	0	0	0	0	0	0	0	0	0	0	0	0	0	0	0	0	0	0	0	0	0	0	0	0	0	0	0	0	0	0
55	141	?	?	?	?	?	?	?	?	?	?	?	?	?	?	?	**?**	?	?	?	?	?	?	?	?	?	?	?	?	?	?	?	?	?	?	?	?	?	?	?	?	?	?	?	?	?	?	?	?	?	?	?	?	?	?	?	?	?	?	?	?	?	?	?	?	?	?	?	?	?	?	?	?	?	?	?	?	0	0	0	0	0
56	129	0	1	1	1	1	1	0	0	0	0	0	0	0	**0**	0	0	0	0	0	0	0	0	0	0	0	0	0	0	0	0	0	0	0	0	0	0	0	0	0	0	0	0	0	0	0	0	0	0	0	0	0	0	0	0	0	0	0	0	0	0	0	0	0	0	0	0	0	0	0	0	0	0	0	0	0	0	0	0	0	0	0
57	156	0	1	1	1	1	1	0	0	0	0	0	0	0	0	0	0	**0**	0	0	0	0	0	0	0	0	0	0	0	0	0	0	0	0	0	0	0	0	0	0	0	0	0	0	0	0	0	0	0	0	0	0	0	0	0	0	0	0	0	0	0	0	0	0	0	0	0	0	0	0	0	0	0	0	0	0	0	0	0	0	0	0
58	155	0	?	?	?	?	?	?	?	?	?	?	?	?	?	?	?	**?**	?	?	?	?	?	?	?	?	?	?	?	?	?	?	?	?	?	?	?	?	?	?	?	?	?	?	?	?	?	?	?	?	?	?	?	?	?	?	?	?	?	?	?	?	?	?	?	?	?	?	?	?	?	?	?	?	?	?	?	0	0	0	0	0
59	154	0	?	?	?	?	?	?	?	?	?	?	?	?	?	?	?	**?**	?	?	?	?	?	?	?	?	?	?	?	?	?	?	?	?	?	?	?	?	?	?	?	?	?	?	?	?	?	?	?	?	?	?	?	?	?	?	?	?	?	?	?	?	?	?	?	?	?	?	?	?	?	?	?	?	?	?	?	0	0	0	0	0
60	152	?	?	?	?	?	?	?	?	?	?	?	?	?	?	?	?	**?**	?	?	?	?	?	?	?	?	?	?	?	?	?	?	?	?	?	?	?	?	?	?	?	?	?	?	?	?	?	?	?	?	?	?	?	?	?	?	?	?	?	?	?	?	?	?	?	?	?	?	?	?	?	?	?	?	?	?	?	0	0	0	0	0
61	151	?	?	?	?	?	?	?	?	?	?	?	?	?	?	?	?	**?**	?	?	?	?	?	?	?	?	?	?	?	?	?	?	?	?	?	?	?	?	?	?	?	?	?	?	?	?	?	?	?	?	?	?	?	?	?	?	?	?	?	?	?	?	?	?	?	?	?	?	?	?	?	?	?	?	?	?	?	0	0	0	0	0
62	154	?	?	?	?	?	?	?	?	?	?	?	?	?	?	?	?	**?**	?	?	?	?	?	?	?	?	?	?	?	?	?	?	?	?	?	?	?	?	?	?	?	?	?	?	?	?	?	?	?	?	?	?	?	?	?	?	?	?	?	?	?	?	?	?	?	?	?	?	?	?	?	?	?	?	?	?	?	0	0	0	0	0
63	157	0	1	1	?	?	?	?	?	?	?	?	?	?	?	?	?	**?**	?	?	?	?	?	?	?	?	?	?	?	?	?	?	?	?	?	?	?	?	?	?	?	?	?	?	?	?	?	?	?	?	?	?	?	?	?	?	?	?	?	?	?	?	?	?	?	?	?	?	?	?	?	?	?	?	?	?	?	0	0	0	0	0
64	151	0	1	1	1	1	?	?	?	?	?	?	?	?	?	?	?	**?**	?	?	?	?	?	?	?	?	?	?	?	?	?	?	?	?	?	?	?	?	?	?	?	?	?	?	?	?	?	?	?	?	?	?	?	?	?	?	?	?	?	?	?	?	?	?	?	?	?	?	?	?	?	?	?	?	?	?	?	0	0	0	0	0
65	164	0	1	1	?	?	?	?	?	?	?	?	?	?	?	?	?	?	**?**	?	?	?	?	?	?	?	?	?	?	?	?	?	?	?	?	?	?	?	?	?	?	?	?	?	?	?	?	?	?	?	?	?	?	?	?	?	?	?	?	?	?	?	?	?	?	?	?	?	?	?	?	?	?	?	?	?	?	0	0	0	0	0
66	161	0	?	?	?	?	?	?	?	?	?	?	?	?	?	?	?	?	**?**	?	?	?	?	?	?	?	?	?	?	?	?	?	?	?	?	?	?	?	?	?	?	?	?	?	?	?	?	?	?	?	?	?	?	?	?	?	?	?	?	?	?	?	?	?	?	?	?	?	?	?	?	?	?	?	?	?	?	0	0	0	0	0
67	116	0	?	?	?	?	?	?	?	?	?	?	?	**?**	?	?	?	?	?	?	?	?	?	?	?	?	?	?	?	?	?	?	?	?	?	?	?	?	?	?	?	?	?	?	?	?	?	?	?	?	?	?	?	?	?	?	?	?	?	?	?	?	?	?	?	?	?	?	?	?	?	?	?	?	?	?	?	0	0	0	0	0

87 **Supplementary Table 5. Maximum likelihood reconstruction table of New World oriole**

88 **annual precipitation niche.** First column shows values obtained using median occurrence value;

89 table cells bolded and boxed indicate which bin encompasses that value. Cell colors indicate bin

90 score; “suitable” (1); dark gray, “unsuitable” (0): white; “unknown” (?): medium gray.

91 Numbered rows indicate reconstructions of ancestral nodes corresponding to numbers in

92 Supplementary Figure 1.

	Median from Occurrence	-10 to 0	1 to 10	11 to 20	21 to 30	31 to 40	41 to 50	51 to 60	61 to 70	71 to 80	81 to 90	91 to 100	101 to 110	111 to 120	121 to 130	131 to 140	141 to 150	151 to 160	161 to 170	171 to 180	181 to 190	191 to 200	201 to 210	211 to 220	221 to 230	231 to 240	241 to 250	251 to 260	261 to 270	271 to 280	281 to 290	291 to 300	301 to 310	311 to 320	321 to 330	331 to 340	341 to 350	351 to 360	361 to 370	371 to 380	381 to 390	391 to 400	401 to 410	411 to 420	421 to 430	431 to 440	441 to 450	451 to 460	461 to 470	471 to 480	481 to 490	491 to 500	501 to 510	511 to 520	521 to 530	531 to 540	541 to 550	551 to 560	561 to 570	571 to 580	581 to 590	591 to 600	601 to 610	611 to 620	621 to 630	631 to 640	641 to 650	651 to 660	661 to 670	671 to 680	681 to 690	691 to 700	701 to 710	711 to 720	721 to 730	731 to 740	741 to 750	751 to 760	761 to 770	771 to 780	781 to 790	791 to 800
Icterus abeillei	166	0	0	0	0	0	0	1	1	1	1	1	1	1	1	1	1	1	**1**	1	1	1	1	1	1	1	1	1	1	1	1	1	1	1	1	1	1	?	?	?	?	?	?	?	?	?	?	?	?	?	?	?	?	?	?	?	?	?	?	?	?	?	?	?	?	?	?	?	?	?	?	?	?	?	?	?	?	?	?	?	?	?
Icterus bullockii	32	1	1	1	1	**1**	1	1	1	1	1	1	1	1	1	1	1	1	1	1	1	1	1	1	1	1	1	1	1	?	?	?	?	?	?	?	?	?	?	?	?	?	?	?	?	?	?	?	?	?	?	?	?	?	?	?	?	?	?	?	?	?	?	?	?	?	?	?	?	?	?	?	?	?	?	?	?	?	?	?	?	?
Icterus cucullatus	6	1	**1**	1	1	1	1	1	1	1	1	1	1	1	1	1	1	1	1	1	1	1	1	1	1	1	1	1	1	1	1	1	1	1	1	1	1	1	1	1	1	1	1	1	1	1	1	1	1	1	1	1	1	1	1	1	1	1	1	1	1	1	1	1	?	?	?	?	?	?	?	?	?	?	?	?	?	?	?	?	?	?
Icterus galbula	113	0	0	0	0	0	1	1	1	1	1	1	1	**1**	1	1	1	1	1	1	1	1	1	1	1	1	0	0	0	0	0	0	0	0	0	0	0	0	0	0	0	0	0	0	0	0	0	0	0	0	0	0	0	0	0	0	0	0	0	0	0	0	0	0	0	0	0	0	0	0	0	0	0	0	0	0	0	0	0	0	0	0	0
Icterus parisorum	50	1	1	1	1	1	**1**	1	1	1	1	1	1	1	1	1	1	1	1	1	1	1	1	1	1	1	1	1	1	?	?	?	?	?	?	?	?	?	?	?	?	?	?	?	?	?	?	?	?	?	?	?	?	?	?	?	?	?	?	?	?	?	?	?	?	?	?	?	?	?	?	?	?	?	?	?	?	?	?	?	?	?
Icterus pustulatus	200	0	0	0	1	1	1	1	1	1	1	1	1	1	1	1	1	1	1	1	1	**1**	1	1	1	1	1	1	1	1	1	1	1	1	1	1	1	1	1	1	1	1	1	1	1	1	1	1	1	1	1	1	1	1	1	1	1	1	1	1	1	1	1	1	1	1	1	1	?	?	?	?	?	?	?	?	?	?	?	?	?	?	?
Icterus spurius	119	0	0	1	1	1	1	1	1	1	1	1	1	1	1	1	1	1	1	1	**1**	1	1	1	1	1	1	1	1	?	?	?	?	?	?	?	?	?	?	?	?	?	?	?	?	?	?	?	?	?	?	?	?	?	?	?	?	?	?	?	?	?	?	?	?	?	?	?	?	?	?	?	?	?	?	?	?	?	?	?	?	?
Icterus auratus	113	?	?	?	?	?	?	1	1	1	1	1	1	**1**	1	1	1	1	0	0	0	0	0	0	0	0	0	0	0	0	0	0	0	0	0	0	0	0	0	0	0	0	0	0	0	0	0	0	0	0	0	0	0	0	0	0	0	0	0	0	0	0	0	0	0	0	0	0	0	0	0	0	0	0	0	0	0	0	0	0	0	0	0
Icterus auricapillus	183	0	0	0	0	0	1	1	1	1	1	1	1	1	1	1	1	1	1	1	**1**	1	1	1	1	1	1	1	1	1	1	1	1	1	1	1	1	1	1	1	1	1	1	1	1	1	1	1	1	?	?	?	?	?	?	?	?	?	?	?	?	?	?	?	?	?	?	?	?	?	?	?	?	?	?	?	?	?	?	?	?	?
Icterus bonana	274	0	0	0	0	0	0	0	0	0	0	0	0	0	0	0	0	0	0	0	1	1	1	1	1	1	1	1	1	**1**	1	1	1	1	?	?	?	?	?	?	?	?	?	?	?	?	?	?	?	?	?	?	?	?	?	?	?	?	?	?	?	?	?	?	?	?	?	?	?	?	?	?	?	?	?	?	?	?	?	?	?	?	?
Icterus cayanensis	236	?	?	?	?	?	?	?	1	1	1	1	1	1	1	1	1	1	1	1	1	1	1	1	1	**1**	1	1	1	1	1	1	1	1	1	1	1	1	1	1	1	1	1	1	1	1	1	?	?	?	?	?	?	?	?	?	?	?	?	?	?	?	?	?	?	?	?	?	?	?	?	?	?	?	?	?	?	?	?	?	?	?
Icterus chrysater	173	0	0	0	0	0	1	1	1	1	1	1	1	1	1	1	1	1	1	**1**	1	1	1	1	1	1	1	1	1	1	1	1	1	1	1	1	1	1	1	1	1	1	1	1	1	1	1	1	1	1	1	1	1	1	1	1	1	1	1	1	1	1	1	1	1	1	1	1	1	1	1	1	1	1	1	1	1	1	1	1	1	?
Icterus chrysocephalus	263	0	0	0	0	0	0	0	0	0	0	0	0	0	0	0	0	1	1	1	1	1	1	1	1	1	1	1	**1**	1	1	1	1	1	1	1	1	1	1	1	1	1	1	1	?	?	?	?	?	?	?	?	?	?	?	?	?	?	?	?	?	?	?	?	?	?	?	?	?	?	?	?	?	?	?	?	?	?	?	?	?	?	?	?
Icterus croconotus	168	0	0	0	0	0	1	1	1	1	1	1	1	1	1	1	1	1	**1**	1	1	1	1	1	1	1	1	1	1	1	1	1	1	1	1	1	1	1	1	1	1	1	1	1	1	1	1	1	1	1	1	1	1	1	1	?	?	?	?	?	?	?	?	?	?	?	?	?	?	?	?	?	?	?	?	?	?	?	?	?	?	?
Icterus dominicensis	130	?	?	?	?	?	?	?	1	1	1	1	1	1	**1**	1	1	1	1	1	1	1	1	1	1	1	?	?	?	?	?	?	?	?	?	?	?	?	?	?	?	?	?	?	?	?	?	?	?	?	?	?	?	?	?	?	?	?	?	?	?	?	?	?	?	?	?	?	?	?	?	?	?	?	?	?	?	?	?	?	?	?	?
Icterus fuertesi	114	0	0	0	0	0	0	0	0	0	1	1	1	**1**	1	1	1	1	1	1	1	0	0	0	0	0	0	0	0	0	0	0	0	0	0	0	0	0	0	0	0	0	0	0	0	0	0	0	0	0	0	0	0	0	0	0	0	0	0	0	0	0	0	0	0	0	0	0	0	0	0	0	0	0	0	0	0	0	0	0	0	0
Icterus graceannae	29	0	1	1	**1**	1	1	1	1	1	1	1	1	1	1	1	0	0	0	0	0	0	0	0	0	0	0	0	0	0	0	0	0	0	0	0	0	0	0	0	0	0	0	0	0	0	0	0	0	0	0	0	0	0	0	0	0	0	0	0	0	0	0	0	0	0	0	0	0	0	0	0	0	0	0	0	0	0	0	0	0	0
Icterus graduacauda	71	?	?	?	1	1	1	1	1	**1**	1	1	1	1	1	1	1	1	1	1	1	1	1	1	1	1	1	1	1	1	1	1	1	1	1	1	1	1	1	1	1	?	?	?	?	?	?	?	?	?	?	?	?	?	?	?	?	?	?	?	?	?	?	?	?	?	?	?	?	?	?	?	?	?	?	?	?	?	?	?	?	?
Icterus gularis	117	0	0	0	0	1	1	1	1	1	1	1	1	**1**	1	1	1	1	1	1	1	1	1	1	1	1	1	1	1	1	1	1	1	1	1	1	1	1	1	1	1	1	1	?	?	?	?	?	?	?	?	?	?	?	?	?	?	?	?	?	?	?	?	?	?	?	?	?	?	?	?	?	?	?	?	?	?	?	?	?	?	?
Icterus icterus	48	?	?	?	?	1	**1**	1	1	1	1	1	1	1	1	1	1	1	1	1	1	1	1	1	1	1	1	1	1	1	1	1	?	?	?	?	?	?	?	?	?	?	?	?	?	?	?	?	?	?	?	?	?	?	?	?	?	?	?	?	?	?	?	?	?	?	?	?	?	?	?	?	?	?	?	?	?	?	?	?	?	?	?
Icterus jamacaii	105	?	?	?	?	1	1	1	1	1	1	1	**1**	1	1	1	1	1	1	1	1	1	1	1	1	?	?	?	?	?	?	?	?	?	?	?	?	?	?	?	?	?	?	?	?	?	?	?	?	?	?	?	?	?	?	?	?	?	?	?	?	?	?	?	?	?	?	?	?	?	?	?	?	?	?	?	?	?	?	?	?	?
Icterus laudabilis	205	?	?	?	?	?	?	?	?	?	?	?	?	?	?	?	?	?	?	?	?	1	**1**	1	?	?	?	?	?	?	?	?	?	?	?	?	?	?	?	?	?	?	?	?	?	?	?	?	?	?	?	?	?	?	?	?	?	?	?	?	?	?	?	?	?	?	?	?	?	?	?	?	?	?	?	?	?	?	?	?	?	?
Icterus leucopteryx	200	?	?	?	?	?	?	?	?	?	?	1	1	1	1	1	1	1	1	1	1	1	**1**	1	1	1	1	1	1	1	1	?	?	?	?	?	?	?	?	?	?	?	?	?	?	?	?	?	?	?	?	?	?	?	?	?	?	?	?	?	?	?	?	?	?	?	?	?	?	?	?	?	?	?	?	?	?	?	?	?	?	?
Icterus maculialatus	136	?	?	?	?	?	?	?	?	?	1	1	1	1	1	**1**	1	1	1	1	1	1	1	1	1	1	1	1	1	1	1	1	1	1	1	1	1	1	1	1	1	1	1	?	?	?	?	?	?	?	?	?	?	?	?	?	?	?	?	?	?	?	?	?	?	?	?	?	?	?	?	?	?	?	?	?	?	?	?	?	?	?
Icterus melanopsis	133	?	?	?	?	?	?	?	?	?	1	1	1	1	1	**1**	1	1	1	1	1	1	1	1	1	1	1	?	?	?	?	?	?	?	?	?	?	?	?	?	?	?	?	?	?	?	?	?	?	?	?	?	?	?	?	?	?	?	?	?	?	?	?	?	?	?	?	?	?	?	?	?	?	?	?	?	?	?	?	?	?	?
Icterus mesomelas	179	0	1	1	1	1	1	1	1	1	1	1	1	1	1	1	1	1	1	**1**	1	1	1	1	1	1	1	1	1	1	1	1	1	1	1	1	1	1	1	1	1	1	1	1	1	1	1	1	1	1	1	1	1	1	1	1	1	1	1	1	1	1	1	1	1	1	1	1	1	1	1	1	1	1	1	1	1	?	?	?	?	?
Icterus nigrogularis	135	?	?	?	1	1	1	1	1	1	1	1	1	1	1	**1**	1	1	1	1	1	1	1	1	1	1	1	1	1	1	1	1	1	1	1	1	1	1	1	1	1	1	1	1	1	1	1	1	1	?	?	?	?	?	?	?	?	?	?	?	?	?	?	?	?	?	?	?	?	?	?	?	?	?	?	?	?	?	?	?	?	?
Icterus northropi	124	?	?	?	?	?	?	?	1	1	1	1	1	1	**1**	?	?	?	?	?	?	?	?	?	?	?	?	?	?	?	?	?	?	?	?	?	?	?	?	?	?	?	?	?	?	?	?	?	?	?	?	?	?	?	?	?	?	?	?	?	?	?	?	?	?	?	?	?	?	?	?	?	?	?	?	?	?	?	?	?	?	?
Icterus oberi	178	?	?	?	?	?	?	?	?	?	?	?	?	?	?	?	?	?	?	**1**	?	?	?	?	?	?	?	?	?	?	?	?	?	?	?	?	?	?	?	?	?	?	?	?	?	?	?	?	?	?	?	?	?	?	?	?	?	?	?	?	?	?	?	?	?	?	?	?	?	?	?	?	?	?	?	?	?	?	?	?	?	?
Icterus pectoralis	162	0	0	0	0	0	0	1	1	1	1	1	1	1	1	1	1	1	**1**	1	1	1	1	1	1	1	1	1	1	1	1	1	1	1	1	1	1	1	1	1	1	1	1	?	?	?	?	?	?	?	?	?	?	?	?	?	?	?	?	?	?	?	?	?	?	?	?	?	?	?	?	?	?	?	?	?	?	?	?	?	?	?
Icterus portoricensis	185	?	?	?	?	?	?	?	?	?	1	1	1	1	1	1	1	1	1	1	**1**	1	1	1	1	1	1	?	?	?	?	?	?	?	?	?	?	?	?	?	?	?	?	?	?	?	?	?	?	?	?	?	?	?	?	?	?	?	?	?	?	?	?	?	?	?	?	?	?	?	?	?	?	?	?	?	?	?	?	?	?	?
Icterus prosthemelas	235	0	0	0	0	0	0	1	1	1	1	1	1	1	1	1	1	1	1	1	1	1	1	1	1	**1**	1	1	1	1	1	1	1	1	1	1	1	1	1	1	1	1	1	1	1	1	1	1	1	1	1	1	1	1	1	1	1	1	?	?	?	?	?	?	?	?	?	?	?	?	?	?	?	?	?	?	?	?	?	?	?	?
Icterus pyrrhopterus	123	?	?	1	1	1	1	1	1	1	1	1	1	1	**1**	1	1	1	1	1	1	1	1	1	1	1	1	1	1	1	1	1	1	1	1	1	1	1	1	1	1	?	?	?	?	?	?	?	?	?	?	?	?	?	?	?	?	?	?	?	?	?	?	?	?	?	?	?	?	?	?	?	?	?	?	?	?	?	?	?	?	?
Icterus wagleri	100	0	0	0	1	1	1	1	1	1	1	**1**	1	1	1	1	1	1	1	1	1	1	1	1	1	1	1	1	1	1	1	1	1	1	1	1	1	1	1	1	1	1	1	?	?	?	?	?	?	?	?	?	?	?	?	?	?	?	?	?	?	?	?	?	?	?	?	?	?	?	?	?	?	?	?	?	?	?	?	?	?	?
35	141	?	?	?	?	?	?	1	1	1	1	1	1	1	1	1	**1**	1	1	1	1	1	1	1	1	1	1	1	1	1	1	1	1	1	1	1	1	1	1	1	1	?	?	?	?	?	?	?	?	?	?	?	?	?	?	?	?	?	?	?	?	?	?	?	?	?	?	?	?	?	?	?	?	?	?	?	?	?	?	?	?	?
36	133	?	?	?	?	?	?	1	1	1	1	1	1	1	1	**1**	1	1	1	1	1	1	1	1	1	1	1	1	1	1	1	1	1	1	1	1	1	1	1	1	1	?	?	?	?	?	?	?	?	?	?	?	?	?	?	?	?	?	?	?	?	?	?	?	?	?	?	?	?	?	?	?	?	?	?	?	?	?	?	?	?	?
37	112	0	?	?	?	1	1	1	1	1	1	1	1	**1**	1	1	1	1	1	1	1	1	1	1	1	1	1	1	1	1	1	1	1	1	1	1	1	1	1	1	1	?	?	?	?	?	?	?	?	?	?	?	?	?	?	?	?	?	?	?	?	?	?	?	?	?	?	?	?	?	?	?	?	?	?	?	?	?	?	?	?	?
38	101	0	?	?	?	1	1	1	1	1	1	1	**1**	1	1	1	1	1	1	1	1	1	1	1	1	1	1	1	1	1	1	1	1	1	1	1	1	1	1	1	1	?	?	?	?	?	?	?	?	?	?	?	?	?	?	?	?	?	?	?	?	?	?	?	?	?	?	?	?	?	?	?	?	?	?	?	?	?	?	?	?	?
39	69	0	0	0	0	1	1	1	**1**	1	1	1	1	1	1	1	1	1	1	1	1	1	1	1	1	1	1	1	1	1	1	1	1	1	1	1	1	1	1	1	1	?	?	?	?	?	?	?	?	?	?	?	?	?	?	?	?	?	?	?	?	?	?	?	?	?	?	?	?	?	?	?	?	?	?	?	?	?	?	?	?	?
40	119	?	?	?	?	1	1	1	1	1	1	1	1	**1**	1	1	1	1	1	1	1	1	1	1	1	1	1	1	1	1	1	1	?	?	?	?	?	?	?	?	?	?	?	?	?	?	?	?	?	?	?	?	?	?	?	?	?	?	?	?	?	?	?	?	?	?	?	?	?	?	?	?	?	?	?	?	?	?	?	?	?	?	?
41	106	?	?	?	?	1	1	1	1	1	1	1	**1**	1	1	1	1	1	1	1	1	1	1	1	1	1	1	1	1	1	1	1	?	?	?	?	?	?	?	?	?	?	?	?	?	?	?	?	?	?	?	?	?	?	?	?	?	?	?	?	?	?	?	?	?	?	?	?	?	?	?	?	?	?	?	?	?	?	?	?	?	?	?
42	137	?	?	?	?	?	?	1	1	1	1	1	1	1	1	**1**	1	1	1	1	1	1	1	1	1	1	1	1	1	1	1	1	1	1	1	1	1	1	1	1	1	?	?	?	?	?	?	?	?	?	?	?	?	?	?	?	?	?	?	?	?	?	?	?	?	?	?	?	?	?	?	?	?	?	?	?	?	?	?	?	?	?
43	136	?	?	?	?	?	?	1	1	1	1	1	1	1	1	**1**	1	1	1	1	1	1	1	1	1	1	1	1	1	1	1	1	1	1	?	?	?	?	?	?	?	?	?	?	?	?	?	?	?	?	?	?	?	?	?	?	?	?	?	?	?	?	?	?	?	?	?	?	?	?	?	?	?	?	?	?	?	?	?	?	?	?	?
44	138	?	?	?	?	?	?	1	1	1	1	1	1	1	1	**1**	1	1	1	1	1	1	1	1	1	1	1	1	1	1	1	?	?	?	?	?	?	?	?	?	?	?	?	?	?	?	?	?	?	?	?	?	?	?	?	?	?	?	?	?	?	?	?	?	?	?	?	?	?	?	?	?	?	?	?	?	?	?	?	?	?	?
45	139	?	?	?	?	?	?	1	1	1	1	1	1	1	1	**1**	1	1	1	1	1	1	1	1	1	1	1	1	1	?	?	?	?	?	?	?	?	?	?	?	?	?	?	?	?	?	?	?	?	?	?	?	?	?	?	?	?	?	?	?	?	?	?	?	?	?	?	?	?	?	?	?	?	?	?	?	?	?	?	?	?	?
46	160	?	?	?	?	?	?	?	1	1	1	1	1	1	1	1	1	**1**	1	1	1	1	1	1	1	1	1	?	?	?	?	?	?	?	?	?	?	?	?	?	?	?	?	?	?	?	?	?	?	?	?	?	?	?	?	?	?	?	?	?	?	?	?	?	?	?	?	?	?	?	?	?	?	?	?	?	?	?	?	?	?	?
47	225	?	?	?	?	?	?	?	1	1	1	1	1	1	1	1	1	1	1	1	1	1	1	1	**1**	1	1	?	?	?	?	?	?	?	?	?	?	?	?	?	?	?	?	?	?	?	?	?	?	?	?	?	?	?	?	?	?	?	?	?	?	?	?	?	?	?	?	?	?	?	?	?	?	?	?	?	?	?	?	?	?	?
48	160	?	?	?	?	?	?	?	1	1	1	1	1	1	1	1	1	**1**	1	1	1	1	1	1	1	1	1	?	?	?	?	?	?	?	?	?	?	?	?	?	?	?	?	?	?	?	?	?	?	?	?	?	?	?	?	?	?	?	?	?	?	?	?	?	?	?	?	?	?	?	?	?	?	?	?	?	?	?	?	?	?	?
49	176	?	?	?	?	?	?	?	1	1	1	1	1	1	1	1	1	1	1	**1**	1	1	1	1	1	1	1	?	?	?	?	?	?	?	?	?	?	?	?	?	?	?	?	?	?	?	?	?	?	?	?	?	?	?	?	?	?	?	?	?	?	?	?	?	?	?	?	?	?	?	?	?	?	?	?	?	?	?	?	?	?	?
50	180	?	?	?	?	?	?	?	?	?	1	1	1	1	1	1	1	1	1	**1**	1	1	1	1	1	1	1	?	?	?	?	?	?	?	?	?	?	?	?	?	?	?	?	?	?	?	?	?	?	?	?	?	?	?	?	?	?	?	?	?	?	?	?	?	?	?	?	?	?	?	?	?	?	?	?	?	?	?	?	?	?	?
51	152	?	?	?	?	?	?	?	1	1	1	1	1	1	1	1	1	**1**	1	1	1	1	1	1	1	1	1	?	?	?	?	?	?	?	?	?	?	?	?	?	?	?	?	?	?	?	?	?	?	?	?	?	?	?	?	?	?	?	?	?	?	?	?	?	?	?	?	?	?	?	?	?	?	?	?	?	?	?	?	?	?	?
52	132	?	?	?	?	?	?	1	1	1	1	1	1	1	1	**1**	1	1	1	1	1	1	1	1	1	1	1	1	1	1	1	1	1	1	1	1	1	1	1	1	1	?	?	?	?	?	?	?	?	?	?	?	?	?	?	?	?	?	?	?	?	?	?	?	?	?	?	?	?	?	?	?	?	?	?	?	?	?	?	?	?	?
53	93	?	?	1	1	1	1	1	1	1	1	**1**	1	1	1	1	1	1	1	1	1	1	1	1	1	1	1	1	1	1	1	1	1	1	1	1	1	1	1	1	1	?	?	?	?	?	?	?	?	?	?	?	?	?	?	?	?	?	?	?	?	?	?	?	?	?	?	?	?	?	?	?	?	?	?	?	?	?	?	?	?	?
54	111	?	?	?	?	?	?	?	1	1	1	1	1	**1**	1	1	1	1	1	1	1	1	1	1	1	1	1	1	1	1	1	1	1	1	1	1	1	1	1	1	1	1	1	1	?	?	?	?	?	?	?	?	?	?	?	?	?	?	?	?	?	?	?	?	?	?	?	?	?	?	?	?	?	?	?	?	?	?	?	?	?	?
55	141	?	?	?	?	?	?	?	1	1	1	1	1	1	1	1	**1**	1	1	1	1	1	1	1	1	1	1	?	?	?	?	?	?	?	?	?	?	?	?	?	?	?	?	?	?	?	?	?	?	?	?	?	?	?	?	?	?	?	?	?	?	?	?	?	?	?	?	?	?	?	?	?	?	?	?	?	?	?	?	?	?	?
56	129	0	0	0	0	0	?	1	1	1	1	1	1	1	**1**	1	1	1	1	1	1	1	1	1	1	1	1	1	1	1	1	1	1	1	1	1	1	1	1	1	1	1	1	1	1	1	1	1	1	?	?	?	?	?	?	?	?	?	?	?	?	?	?	?	?	?	?	?	?	?	?	?	?	?	?	?	?	?	?	?	?	?
57	156	0	0	0	0	0	?	1	1	1	1	1	1	1	1	1	1	**1**	1	1	1	1	1	1	1	1	1	1	1	?	?	?	?	?	?	?	?	?	?	?	?	?	?	?	?	?	?	?	?	?	?	?	?	?	?	?	?	?	?	?	?	?	?	?	?	?	?	?	?	?	?	?	?	?	?	?	?	?	?	?	?	?
58	155	0	0	0	1	1	1	1	1	1	1	1	1	1	1	1	1	**1**	1	1	1	1	1	1	1	1	1	1	1	1	1	1	1	1	1	1	1	1	1	1	1	?	?	?	?	?	?	?	?	?	?	?	?	?	?	?	?	?	?	?	?	?	?	?	?	?	?	?	?	?	?	?	?	?	?	?	?	?	?	?	?	?
59	154	0	0	0	1	1	1	1	1	1	1	1	1	1	1	1	1	**1**	1	1	1	1	1	1	1	1	1	1	1	1	1	1	1	1	1	1	1	1	1	1	1	?	?	?	?	?	?	?	?	?	?	?	?	?	?	?	?	?	?	?	?	?	?	?	?	?	?	?	?	?	?	?	?	?	?	?	?	?	?	?	?	?
60	152	?	?	?	?	1	1	1	1	1	1	1	1	1	1	1	1	**1**	1	1	1	1	1	1	1	1	1	1	1	1	1	1	1	1	1	1	1	1	1	1	1	?	?	?	?	?	?	?	?	?	?	?	?	?	?	?	?	?	?	?	?	?	?	?	?	?	?	?	?	?	?	?	?	?	?	?	?	?	?	?	?	?
61	151	?	?	?	?	1	1	1	1	1	1	1	1	1	1	1	1	**1**	1	1	1	1	1	1	1	1	1	1	1	1	1	1	1	1	1	1	1	1	1	1	1	?	?	?	?	?	?	?	?	?	?	?	?	?	?	?	?	?	?	?	?	?	?	?	?	?	?	?	?	?	?	?	?	?	?	?	?	?	?	?	?	?
62	154	?	?	?	1	1	1	1	1	1	1	1	1	1	1	1	1	**1**	1	1	1	1	1	1	1	1	1	1	1	1	1	1	1	1	1	1	1	1	1	1	1	?	?	?	?	?	?	?	?	?	?	?	?	?	?	?	?	?	?	?	?	?	?	?	?	?	?	?	?	?	?	?	?	?	?	?	?	?	?	?	?	?
63	157	0	0	0	1	1	1	1	1	1	1	1	1	1	1	1	1	**1**	1	1	1	1	1	1	1	1	1	1	1	1	1	1	1	1	1	1	1	?	?	?	?	?	?	?	?	?	?	?	?	?	?	?	?	?	?	?	?	?	?	?	?	?	?	?	?	?	?	?	?	?	?	?	?	?	?	?	?	?	?	?	?	?
64	151	0	0	0	0	0	?	1	1	1	1	1	1	1	1	1	1	**1**	1	1	1	1	1	1	1	1	1	1	1	1	1	1	1	1	1	1	1	?	?	?	?	?	?	?	?	?	?	?	?	?	?	?	?	?	?	?	?	?	?	?	?	?	?	?	?	?	?	?	?	?	?	?	?	?	?	?	?	?	?	?	?	?
65	164	0	0	0	1	1	1	1	1	1	1	1	1	1	1	1	1	1	**1**	1	1	1	1	1	1	1	1	1	1	1	1	1	1	1	1	1	1	?	?	?	?	?	?	?	?	?	?	?	?	?	?	?	?	?	?	?	?	?	?	?	?	?	?	?	?	?	?	?	?	?	?	?	?	?	?	?	?	?	?	?	?	?
66	161	1	1	1	1	1	1	1	1	1	1	1	1	1	1	1	1	1	**1**	1	1	1	1	1	1	1	1	1	1	1	1	1	1	1	1	1	1	1	1	1	1	?	?	?	?	?	?	?	?	?	?	?	?	?	?	?	?	?	?	?	?	?	?	?	?	?	?	?	?	?	?	?	?	?	?	?	?	?	?	?	?	?
67	116	0	0	?	?	?	?	1	1	1	1	1	1	**1**	1	1	1	1	1	1	1	1	1	1	1	1	1	1	1	1	1	1	1	1	1	1	1	1	1	1	1	?	?	?	?	?	?	?	?	?	?	?	?	?	?	?	?	?	?	?	?	?	?	?	?	?	?	?	?	?	?	?	?	?	?	?	?	?	?	?	?	?

93

94
